# Biochemical analysis of leishmanial and human GDP-Mannose Pyrophosphorylases and selection of inhibitors as new leads

**DOI:** 10.1038/s41598-017-00848-8

**Published:** 2017-04-07

**Authors:** Wei Mao, Pierre Daligaux, Noureddine Lazar, Tâp Ha-Duong, Christian Cavé, Herman van Tilbeurgh, Philippe M. Loiseau, Sébastien Pomel

**Affiliations:** 1 Chimiothérapie Antiparasitaire, UMR CNRS 8076 BioCIS, Université Paris-Sud, Université Paris-Saclay, 5 rue Jean-Baptiste Clément, 92290 Châtenay-Malabry, France; 2 Institut de Biologie Intégrative de la Cellule, UMR 9198, CNRS, Université Paris-Sud, Université Paris-Saclay, Bâtiment 430, Orsay, 91405 France; 3 Molécules Fluorées et Chimie Médicinale, UMR CNRS 8076 BioCIS, Université Paris-Sud, Université Paris-Saclay, 5 rue Jean-Baptiste Clément, 92290 Châtenay-Malabry, France

## Abstract

Leishmaniases are an ensemble of diseases caused by the protozoan parasite of the genus *Leishmania*. Current antileishmanial treatments are limited and present main issues of toxicity and drug resistance emergence. Therefore, the generation of new inhibitors specifically directed against a leishmanial target is an attractive strategy to expand the chemotherapeutic arsenal. GDP-Mannose Pyrophosphorylase (GDP-MP) is a prominent therapeutic target involved in host-parasite recognition which has been described to be essential for parasite survival. In this work, we produced and purified GDP-MPs from *L. mexicana* (*Lm*GDP-MP), *L. donovani* (*Ld*GDP-MP), and human (*h*GDP-MP), and compared their enzymatic properties. From a rationale design of 100 potential inhibitors, four compounds were identified having a promising and specific inhibitory effect on parasite GDP-MP and antileishmanial activities, one of them exhibits a competitive inhibition on *Ld*GDP-MP and belongs to the 2-substituted quinoline series.

## Introduction

Leishmaniases are Neglected Tropical Diseases (NTD) caused by protozoan parasites belonging to the genus *Leishmania*, and transmitted by the female sandfly. Currently, this NTD threatens 310 million people in 98 countries with 1.3 million new cases per year^1^. There are three main clinical manifestations of leishmaniasis: cutaneous (CL), which causes skin sores and chronic ulcers, muco-cutaneous (MCL), affecting naso-oro-pharyngeal mucosa and causing facial disfigurement, and visceral (VL) which is lethal without treatment. *L. mexicana* and *L. donovani* are two leishmanial species distributed in the New and Old World and responsible of CL and VL, respectively.

The existing treatments suggested by World Health Organization are mainly based on the use of antimonials, liposomal amphotericin B, miltefosine and paromomycin^2^. All of them having main issues of toxicity and drug resistance emergence^3^, the development of new inhibitors specifically directed against a parasite target is an attractive approach for identifying new drugs. The GDP-mannose Pyrophosphorylase (GDP-MP) is an attractive therapeutic target due to its essential role in amastigote survival in macrophages both *in vitro* and *in vivo* and its involvement in the biosynthesis of diverse glycoconjugates necessary for host cell recognition^4, 5^. Furthermore, by molecular modeling, we identified several structural differences between the parasite and the human orthologs, including the presence of a specific motif in the catalytic pocket, making this enzyme a target of choice for the development of new specific antileishmanial agents^6, 7^. A high throughput screening performed on *L. major* GDP-MP allowed the identification of a quinoline derivative, presenting an IC_50_ value on the enzyme at the submicromolar range and on intramacrophage parasites at about 20 µM^8^. Various quinoline derivatives have been identified exhibiting antileishmanial activity^9^. Our laboratory has previously revealed the 2-substituted quinoline series as antileishmanial lead^10, 11^. Thus, in the present study, we decided to integrate this series in the design of GDP-MP competitive inhibitors. Preliminary molecular modeling studies allowed us to hypothesize that the quinoline motif could replace the guanine group of GDP-mannose within the GDP-MP catalytic site. Therefore, GDP-MP competitive inhibitors could be designed by including such quinoline group in the inhibitor scaffold. Various pharmacomodulations were also carried out without quinoline, supplying an in-house library of 100 compounds that have been studied in the present work.

Starting from mannose-1-phosphate (Man-1-P) and GTP, GDP-MP catalyzes the formation of GDP-mannose. This activated form of mannose is a key substrate for different glycosylation processes such as N-glycosylation or O-mannosylation which are essential for post-translational modifications in eukaryotes^12^. In *Leishmania sp*., GDP-mannose is a precursor for the synthesis of a range of mannose containing glycoconjugates involved in host cell recognition^13^. Many GDP-MPs appear to be dimeric, like in bacteria and in some protozoa such as *Trypanosoma brucei*, and in some bacterial species, the protein presents both GDP-MP and phosphomannose isomerase activities, leading to bifunctional enzymes^14–17^. In mammalian, GDP-MP is composed of two subunits α and β, the latter containing the GDP-MP catalytic activity *per se*, while the former would have a regulatory role allowing allosteric inhibition of the β subunit by GDP-mannose^18–20^. In human, the gene encoding for GDP-MP β subunit is transcribed as two isoforms β1 and β2. In opposition to β1, β2 isoform is strongly expressed within a wide tissue distribution. Unlike in other organisms, in *L. mexicana*, GDP-MP is a hexamer which can dissociate in trimers and monomers in low ionic strength conditions leading to a mixture of the three forms^21^. Although the GDP-MP activity was detected in the size range of a hexamer (240 kDa), the trimer and monomer forms were not described to be inactive.

In this study, we compared enzymatic parameters of GDP-MPs from two geographically distant leishmanial species, *L. donovani* (*Ld*GDP-MP), found in Africa and Asia, and *L. mexicana* (*Lm*GDP-MP), encountered in South America, with those from the human ortholog (*h*GDP-MP), with the objective to select specific inhibitors of leishmanial GDP-MPs. These compounds rationally designed from docking analysis were evaluated on the purified enzymes as well as on both parasite species *in vitro*.

## Results

### Purification of GDP-MPs

**Figure 1:**
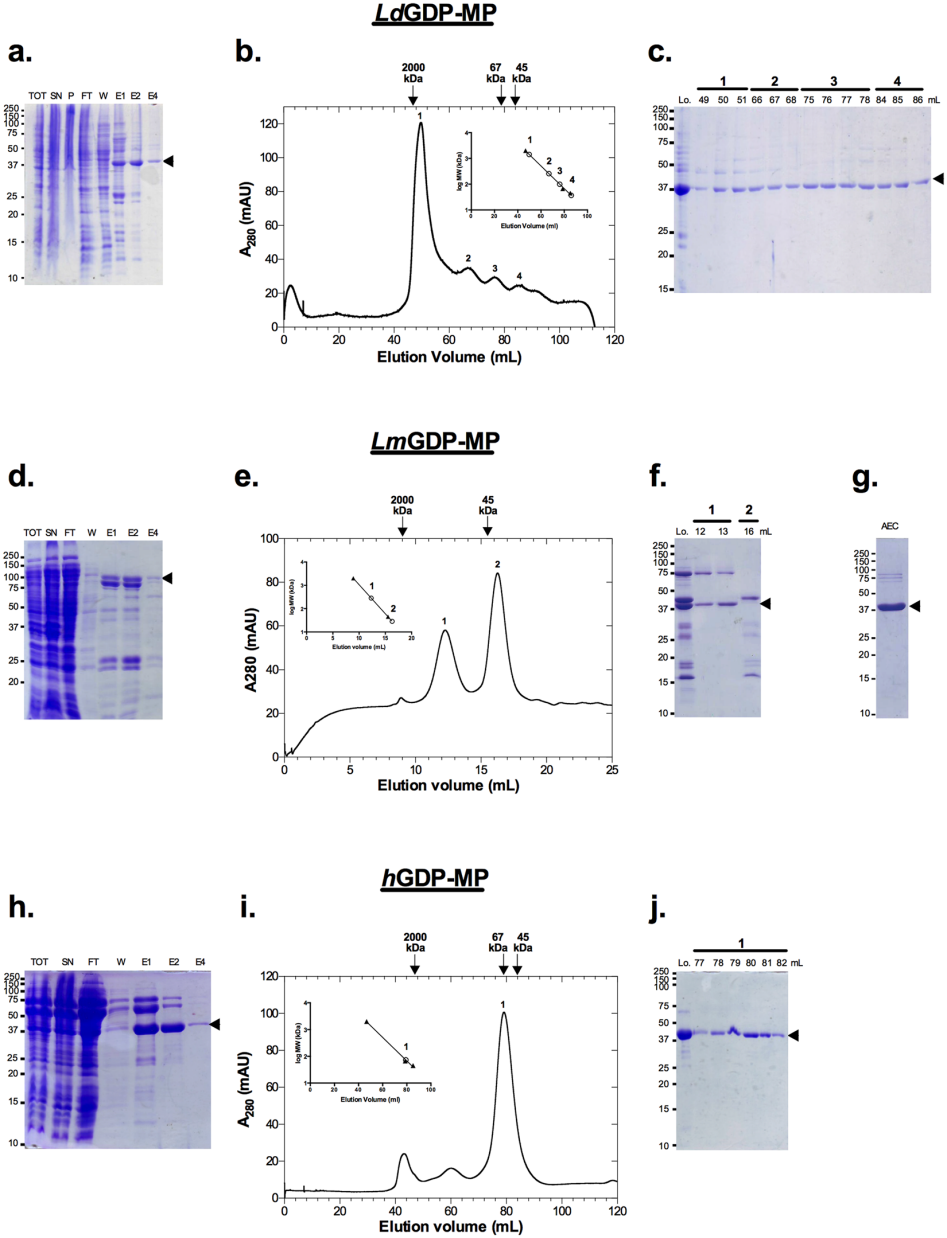
Purification of *Ld*GDP-MP, *Lm*GDP-MP and *h*GDP-MP. (**a,d,h**) Coomassie blue stained SDS-PAGE of *Ld*GDP-MP (**a**), *Lm*GDP-MP (**d**), and *h*GDP-MP (**h**) purified from Ni-NTA column. Lane TOT: total, Lane SN: supernatant, Lane P: Pellet, Lane FT: flow through, Lane W: wash, Lanes E1, E2 and E4: elution fraction at 100 µM, 200 µM and 400 µM imidazole, respectively. The arrowheads show the proteins of interest. The protein ladders on the left are indicated in kDa. Full length gels are presented in Supplementary Fig. S7. (**b,e,i**) Size exclusion chromatography profiles of *Ld*GDP-MP (**b**), *Lm*GDP-MP (**e**), and *h*GDP-MP (**i**). In the inset, the filled triangles and open circles represent standard proteins and the peaks of proteins of interest, respectively. (**c,f,j**) Coomassie blue stained SDS-PAGE of fraction volumes of peaks 1 (49–51 mL), 2 (66–68 mL), 3 (75–78 mL) and 4 (84–86 mL) of *Ld*GDP-MP (**c**), peaks 1 (12–13 mL) and 2 (16 mL) of *Lm*GDP-MP (**f**) and peak 1 (77–82 mL) of *h*GDP-MP (**j**). Lo is the loading control. The arrowheads show the proteins of interest. The protein ladder on the left is indicated in kDa. Full length gels are presented in Supplementary Fig. S7. (**g**) Coomassie blue stained SDS-PAGE of a concentrated pool of fractions containing *Lm*GDP-MP eluted from anion exchange chromatography (AEC). The arrowhead shows the protein of interest. The protein ladder on the left is indicated in kDa. Full length gel is presented in Supplementary Fig. S7.

Although *L. mexicana* and *L. donovani* are geographically distant parasite species, *Lm*GDP-MP and *Ld*GDP-MP share 94% identity in sequence. The human GDP-MP isoform analyzed in this work is the β2 subunit (*h*GDP-MP), which presents a wide tissue distribution, and has a higher identity score for the leishmanial counterparts (∼50%), compared to the β1 subunit (∼46%). While amino acids involved in substrate binding are conserved between *Lm*GDP-MP and *Ld*GDP-MP, several differences have been identified in the active site of *h*GDP-MP^6, 7^. In order to compare their enzymatic properties, His6-tagged *Ld*GDP-MP, *Lm*GDP-MP and *h*GDP-MP were produced and purified using nickel affinity chromatography (Fig. 1a,d and h). *Ld*GDP-MP was further purified by size exclusion chromatography (Fig. 1b). The profile of gel filtration shows one main peak at 50 mL and three smaller peaks at 67, 76 and 86 mL. As revealed in SDS-PAGE, the fractions from peaks 1 to 4 showed the presence of a markedly major band at 42 kDa, corresponding to the predicted molecular weight of *Ld*GDP-MP and showing 63% sequence coverage with *Ld*GDP-MP in mass spectrometry (MS; Fig. 1c, Supplementary Fig. S1). The estimated molecular weight of the peak 1 at around 2,000 kDa (Fig. 1b and Supplementary Table S1) indicates that *Ld*GDP-MP is an aggregated soluble form in these fractions. Major and comparable GDP-MP activities were measured in peaks 2, 3 and 4 whose elution volumes correspond to hexameric (240 kDa), trimeric (120 kDa) and monomeric (40 kDa) forms of the enzyme, respectively. The fractions of peaks 2, 3 and 4 were further pooled and concentrated for Dynamic Light Scattering analysis (Table S1). The size of the objects detected was estimated at 240 kDa with a low polydispersity, showing that *Ld*GDP-MP is mainly in the hexameric form in this concentrated pool of fractions.

Following purification on a nickel affinity column, His6-MBP tagged *Lm*GDP-MP was further purified by anion exchange chromatography. The purified fusion protein of 84 kDa was digested by TEV to remove the tag and loaded on a gel filtration column. The elution profile shows two peaks containing either two bands of 41 and 75 kDa or one main band of 43 kDa, as revealed by SDS-PAGE (Fig. 1e and f). The 43 kDa band corresponding to the predicted molecular weight of His6-MBP tag, and the MS analysis of the 41 kDa band revealing 41% sequence coverage with *Lm*GDP-MP (Supplementary Fig. S1), gel filtration allowed to separate efficiently *Lm*GDP-MP from His6-MBP tag. Thereafter, the contaminant at 75 kDa was mostly removed by anion exchange chromatography, as revealed in Fig. 1g. As opposed to *Ld*GDP-MP, *Lm*GDP-MP was eluted in a single gel filtration peak (peak 1 in Fig. 1e), with an estimated molecular weight of 240 kDa corresponding with a hexameric form of the protein.

Three steps of purification were used to purify *h*GDP-MP: nickel affinity, anion exchange and gel filtration chromatographies. The elution profile of the last step shows that *h*GDP-MP is eluted in one main peak corresponding to the dimeric form of *h*GDP-MP (Fig. 1i). The analysis of the fractions of this peak in SDS-PAGE revealed a single band at 39 kDa which showed 63% sequence coverage with *h*GDP-MP in MS (Fig. 1j and Supplementary Fig. S1).

Fractions corresponding to purified *Ld*GDP-MP (peaks 2, 3 and 4 in Fig. 1c), *Lm*GDP-MP (Fig. 1g) and *h*GDP-MP (Fig. 1j) were pooled and concentrated for further enzymatic studies.

### Enzymatic properties

**Figure 2:**
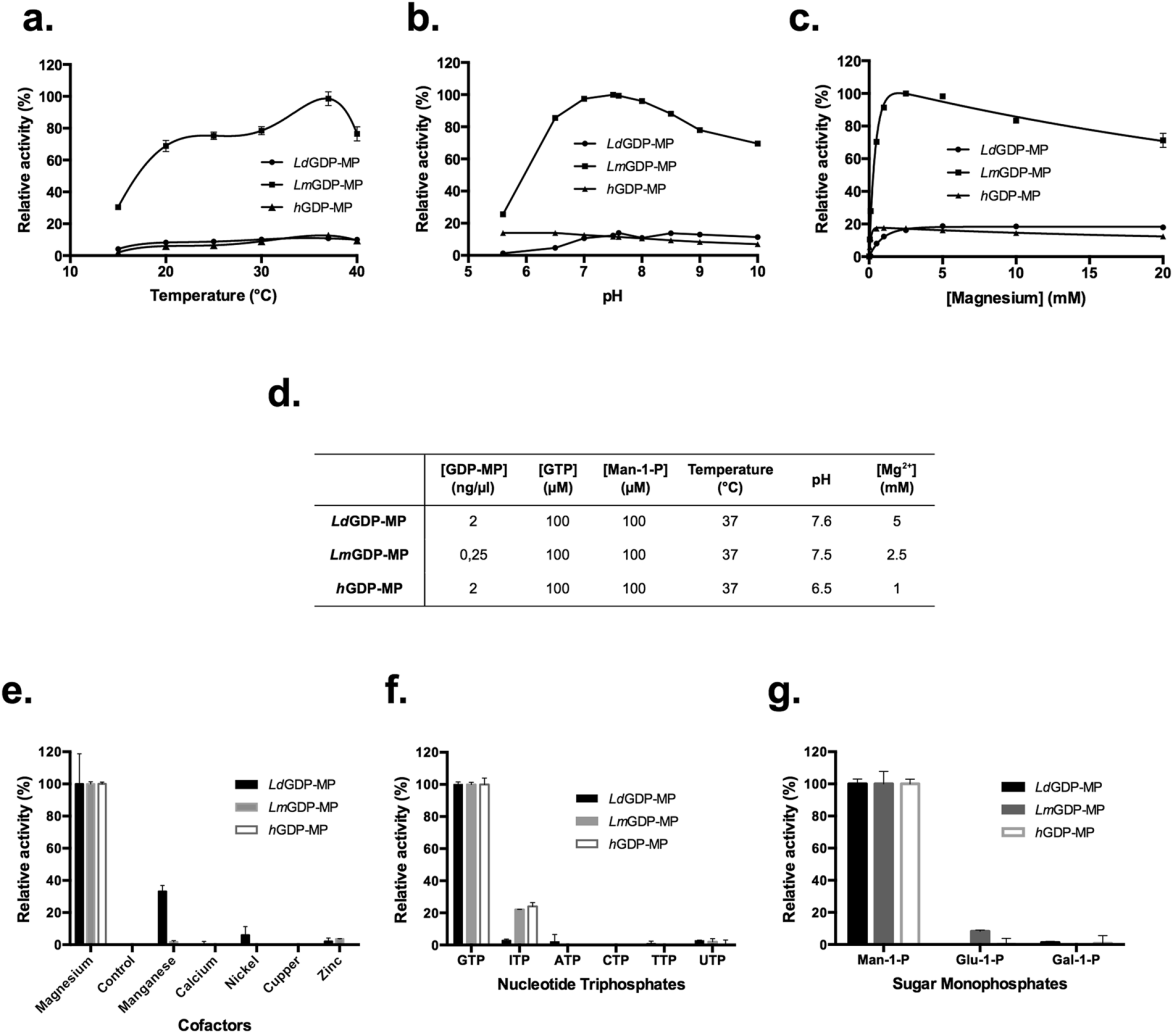
Determination of *Ld*GDP-MP, *Lm*GDP-MP and *h*GDP-MP optimal conditions of enzymatic reaction and specificities. (**a,b,c**) Determination of *Ld*GDP-MP, *Lm*GDP-MP and *h*GDP-MP optimal temperatures (**a**), pH (**b**) and Mg^2+^ concentrations (**c**). For the determination of the optimal pH, the buffers used are 50 mM MES for pH 5.6 and 6.5, and 50 mM Tris-HCl for pH between 6.8 and 10. In each graph, enzyme activities were calculated relative to the maximal specific activity obtained with *Lm*GDP-MP and are expressed in percent. The results correspond to the mean of three independent experiments ± SD. (**d**) Optimal parameters of *Ld*GDP-MP, *Lm*GDP-MP and *h*GDP-MP enzymatic reactions. All reactions were performed with the optimal reaction time of 20 min. (**e,f,g**) Determination of *Ld*GDP-MP, *Lm*GDP-MP and *h*GDP-MP specificities for cofactors (**e**), nucleotide triphosphates (**f**) or sugar monophosphates (**g**). All cofactors (Mg^2+^, Mn^2+^, Ca^2+^, Ni^2+^, Cu^2+^, Zn^2+^) were used at 5 mM. The NTPs (GTP, ATP, CTP, TTP, UTP and ITP) and sugar monophosphates (Man-1-P, Glu-1-P and Gal-1-P) were used at 100 µM. Enzyme activities were calculated relative to each enzyme specific activity obtained with Mg^2+^ (**e**), GTP (**f**) or Man-1-P (**g**), and are expressed in percent. The results correspond to the mean of three independent experiments ± SD.

As shown in Fig. 2a and Supplementary Fig. S3, the best reaction conditions were obtained at 37 °C with a 20 min reaction time for the three purified GDP-MPs. From Michaelis-Menten plots, the optimal concentrations of Man-1-P and GTP have been determined at 100 µM for the three GDP-MPs, although enzyme saturation was already reached at 50 µM of both substrates (Supplementary Fig. S5), and a significant inhibition was observed with a concentration higher than 150 µM of GTP, but not Man-1-P (data not shown). Enzyme concentration was reduced by a factor 8 (0.25 ng/µl) for *Lm*GDP-MP compared to the two other GDP-MPs in order to stay in the linear regime of the detection assay (Supplementary Fig. S4). The pH optima for *Ld*GDP-MP and *Lm*GDP-MP are similar (pH 7.5 and 7.6), and slightly more acidic for *h*GDP-MP (pH 6.5; Fig. 2b). In these optimal conditions, the activities of *Ld*GDP-MP and *h*GDP-MP are only 15–20% relative to that of *Lm*GDP-MP (Fig. 2a–c). In absence of Mg^2+^, no enzyme activity was observed, showing this cofactor is necessary for the activity of the three purified GDP-MPs (Fig. 2e). Optimal concentrations of Mg^2+^ were determined at 1 mM, 2.5 mM and 5 mM for *h*GDP-MP, *Lm*GDP-MP and *Ld*GDP-MP, respectively (Fig. 2c). No GDP-MP activity was observed with any other divalent ions, such as Mn^2+^, Ca^2+^, Ni^2+^, Cu^2+^ and Zn^2+^, except for Mn^2+^ which can be used as a cofactor by *Ld*GDP-MP to generate 33% of activity compared to Mg^2+^ (Fig. 2e). Likewise, no GDP-MP activity was observed with substrate analogues, except for ITP which can be used by *Lm*GDP-MP or *h*GDP-MP to generate 22–24% of activity compared to GTP (Fig. 2f and g). These results show that the three purified GDP-MPs present high cofactor and substrate specificities. All further enzymatic analyses were performed using the optimal conditions described in Fig. 2d.

**Figure 3:**
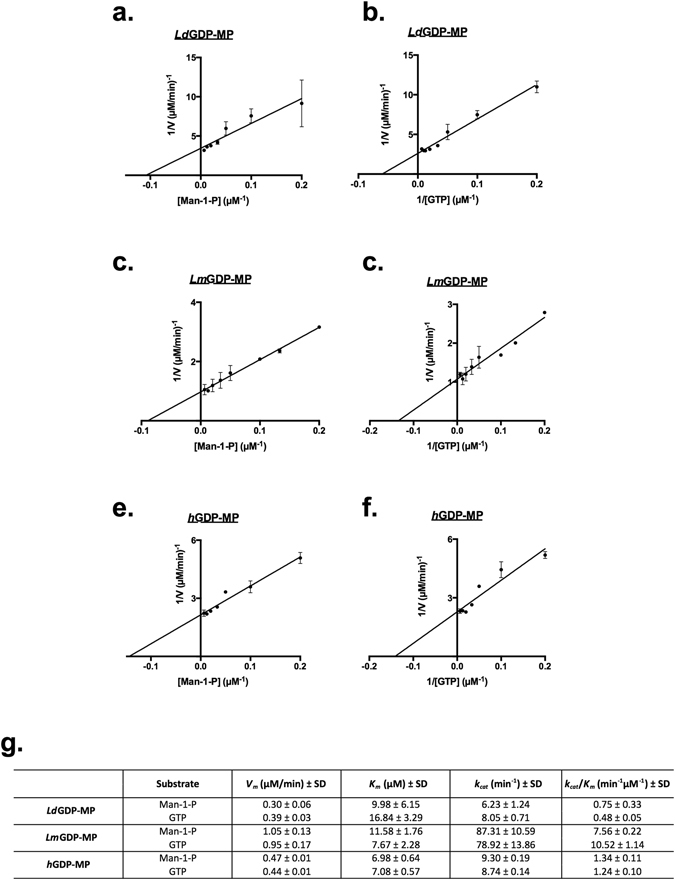
Determination of *Ld*GDP-MP, *Lm*GDP-MP and *h*GDP-MP kinetic constants. (**a,c,e**) Lineweaver Burk double reciprocal plots 1/V = f(1/[Man-1-P]) of *Ld*GDP-MP (**a**), *Lm*GDP-MP (**c**) and *h*GDP-MP (**e**). GTP concentration was held constant at 150 µM for *Ld*GDP-MP and 80 µM for *Lm*GDP-MP and *h*GDP-MP. The results expressed correspond to the mean of three independent experiments ± SD. (**b,d,f**) Lineweaver Burk double reciprocal plots 1/V = f(1/[GTP]) of *Ld*GDP-MP (**b**), *Lm*GDP-MP (**d**) and *h*GDP-MP (**f**). Man-1-P concentration was held constant at 150 µM for *Ld*GDP-MP and 80 µM for *Lm*GDP-MP and *h*GDP-MP. The results expressed correspond to the mean of three independent experiments ± SD. (**g**) Kinetic constants (*V*_*m*_, *K*_*m*_, *k*_*cat*_, *k*_*cat*_/*K*_*m*_) of *Ld*GDP-MP, *Lm*GDP-MP and *h*GDP-MP for both substrates Man-1-P and GTP.

Kinetic constants of each purified GDP-MP for both substrates, GTP and Man-1-P, were determined by using non-linear regression analysis (Fig. 3g) and graphical representations were depicted using Lineweaver-Burk double reciprocal plots (Fig. 3a–f). The maximal velocities (*V*_*m*_) of *Ld*GDP-MP and *h*GDP-MP have comparable values for GTP and Man-1-P ranging from 0.30 to 0.47 µM/min, while the *Lm*GDP-MP *V*_*m*_ value is two to three times higher for both substrates. Accordingly, *Lm*GDP-MP exhibits a turnover rate (*k*_*cat*_) 10 times higher than the two other purified enzymes, with a value at around 80 min^−1^ for both substrates. The *K*_*m*_ values of *h*GDP-MP is at around 7 µM for both substrates and is comparable or slightly lower than the *K*_*m*_ values obtained with leishmanial GDP-MPs, reflecting a moderate higher affinity of *h*GDP-MP for both substrates compared to leishmanial GDP-MPs. The *K*_*m*_ values of *Lm*GDP-MP are 11.58 ± 1.76 µM for Man-1-P and 7.67 ± 2.28 µM for GTP suggesting it has a higher affinity for GTP than for Man-1-P. This result is in opposition to *Ld*GDP-MP which presents a smaller *K*_*m*_ for Man-1-P compared to GTP. The calculated catalytic efficiencies of *Lm*GDP-MP (*k*_*cat*_/*K*_*m*_) at 7.56 ± 0.22 min^−1^ µM^−1^ and 10.52 ± 1.14 min^−1^ µM^−1^ are also 10 to 20 times higher than for the two other GDP-MPs. In addition, the catalytic efficiency of *h*GDP-MP is similar for Man-1-P and GTP at around 1.3 min^−1^ µM^−1^, and is twice higher than the *k*_*cat*_/*K*_*m*_ calculated for *Ld*GDP-MP for both substrates.

**Figure 4:**
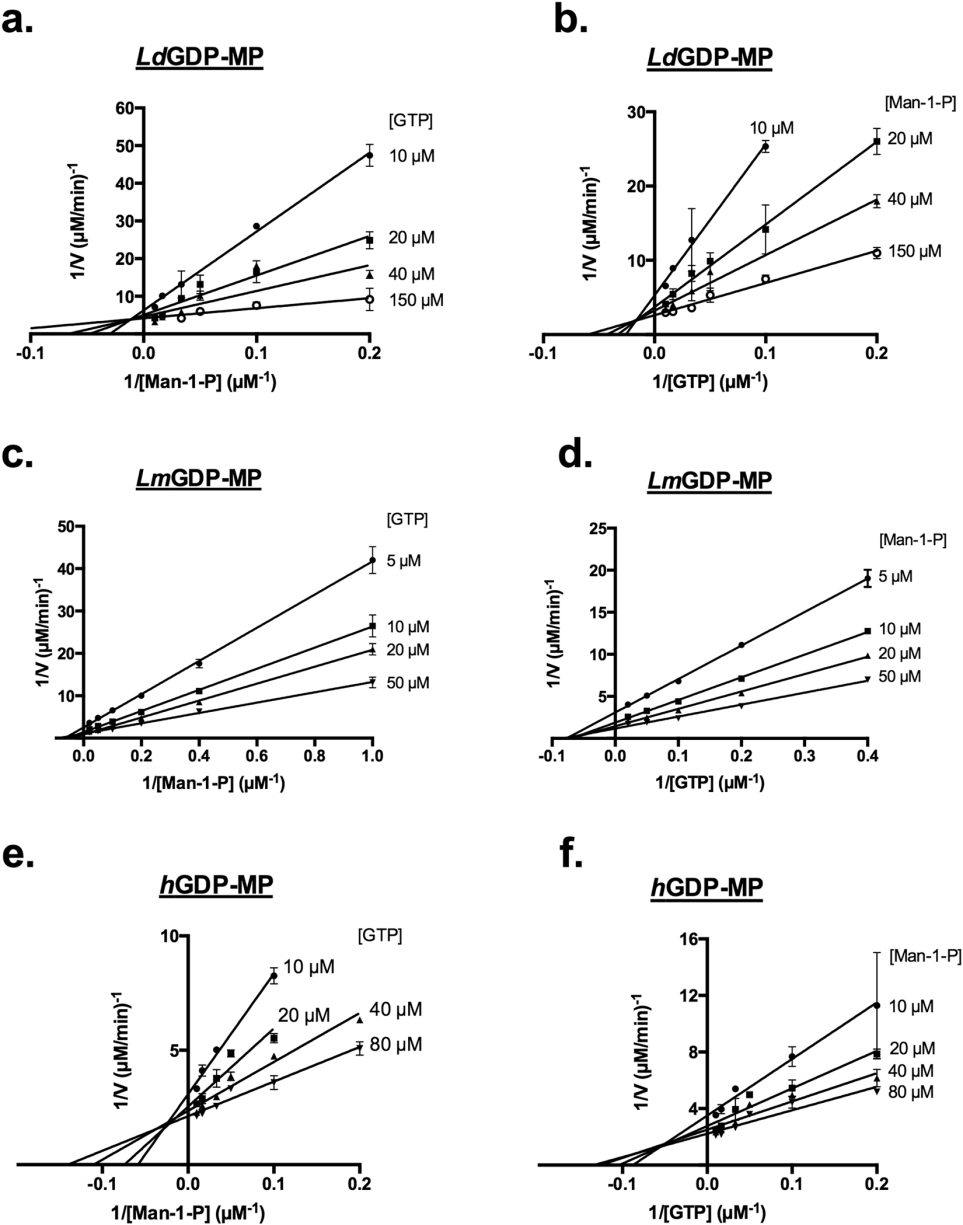
Determination of *Ld*GDP-MP, *Lm*GDP-MP and *h*GDP-MP mechanisms of reaction. (**a** and **b**) Lineweaver Burk plots 1/V = f(1/[Man-1-P]) (**a**) and 1/V = f(1/[GTP]) (**b**) of *Ld*GDP-MP. Constant concentrations of GTP (**a**) and Man-1-P (**b**) were held at 10 µM, 20 µM, 40 µM and 150 µM. The results expressed correspond to the mean of three independent experiments ± SD. (**c** and **d**) Lineweaver Burk plots 1/V = f(1/[Man-1-P]) (**c**) and 1/V = f(1/[GTP]) (**d**) of *Lm*GDP-MP. Constant concentrations of GTP (**c**) and Man-1-P (**d**) were held at 5 µM, 10 µM, 20 µM and 50 µM. The results expressed correspond to the mean of three independent experiments ± SD. (**e** and **f**) Lineweaver Burk plots 1/V = f(1/[Man-1-P]) (**e**) and 1/V = f(1/[GTP]) (**f**) of *h*GDP-MP. Constants concentrations of GTP (**e**) and Man-1-P (**f**) were held at 10 µM, 20 µM, 40 µM and 80 µM. The results expressed correspond to the mean of three independent experiments ± SD.

In order to investigate the mechanism of reaction of each purified GDP-MPs and to assess the kinetics of substrates binding, enzyme activities were analyzed with a range of Man-1-P concentrations and a fixed GTP concentration (Fig. 4a,c and e), and inversely with a range of GTP concentrations and a fixed Man-1-P concentration (Fig. 4b,d and f). In these two series of enzyme assays the double inverse plots intersect on the left of the 1/y axis for the three GDP-MPs (Fig. 4), which is consistent with the formation of a ternary complex, and thus a sequential mechanism^22^. Furthermore, for both substrates, the intersection of double inverse plots is above the 1/x axis for *Ld*GDP-MP and *h*GDP-MP, and on the 1/x axis for *Lm*GDP-MP (Fig. 4a,b,e and f). These analyses support a sequential random mechanism for the three enzymes. Moreover, for *Ld*GDP-MP and *h*GDP-MP, the fixation of one substrate would be favored by the binding of the other. For *Lm*GDP-MP, both substrates would bind sequentially to the enzyme, but independently from one another.

### Selection of leishmanial GDP-MP inhibitors

**Figure 5:**
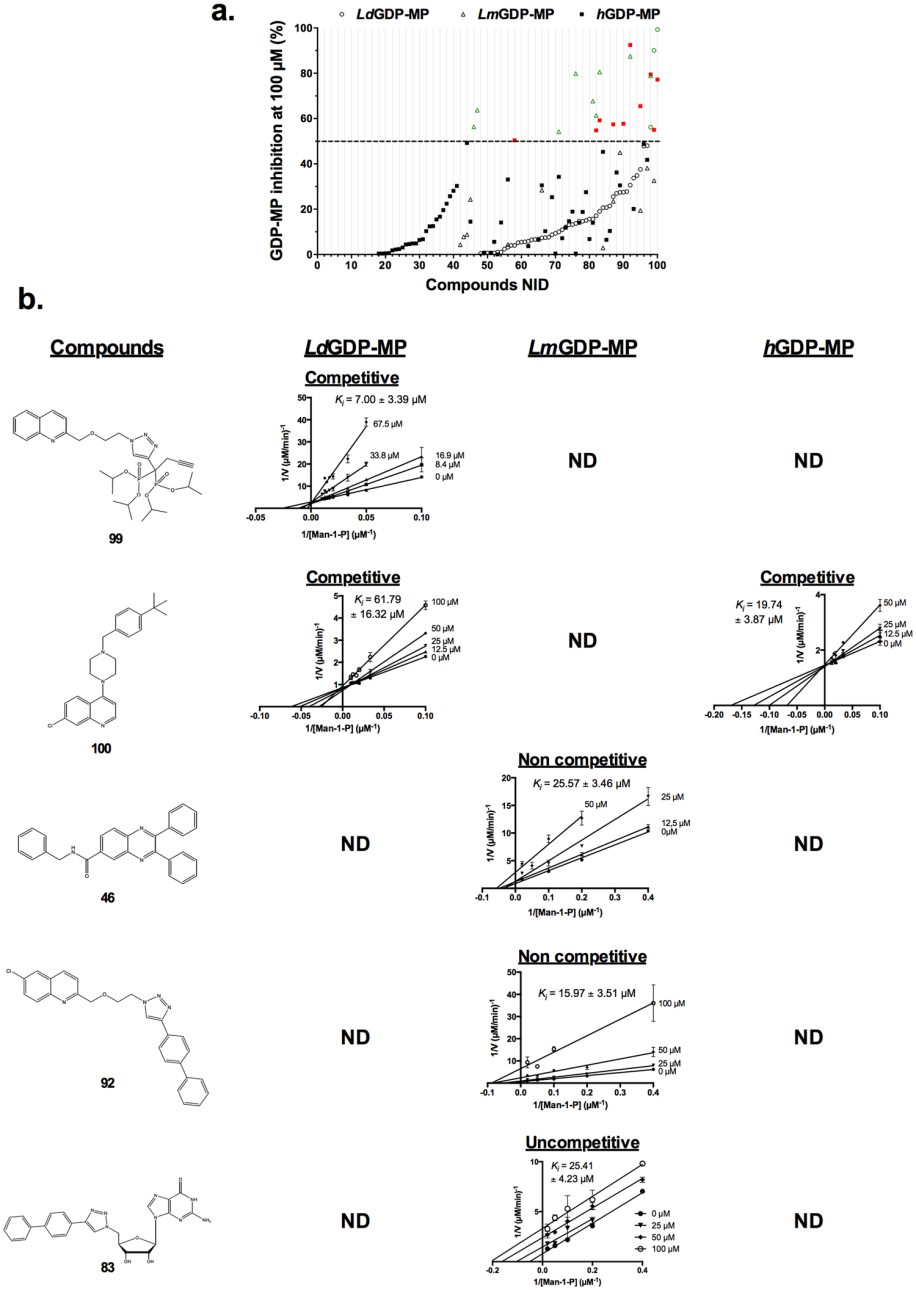
Evaluation of compound activities on *Ld*GDP-MP, *Lm*GDP-MP and *h*GDP-MP. (**a**) Percentage of *Ld*GDP-MP (Ο), *Lm*GDP-MP (Δ) and *h*GDP-MP (■) inhibition as a function of compounds numerical ID (NID). Each compound was used at 100 µM. Compounds showing inhibition above 50% are represented in green and red for leishmanial and human GDP-MPs, respectively. For a question of readability, all values at 0% inhibition were removed. The results expressed correspond to the mean of three independent experiments. (**b**) Lineweaver plots double reciprocal plots 1/V = f(1/[Man-1-P]) of compounds **99**, **100**, **46**, **92** and **83** on *Ld*GDP-MP*, Lm*GDP-MP and *h*GDP-MP. Each compound was evaluated with a range of concentrations: 0–67.5 µM for compound **99** on *Ld*GDP-MP, 0–50 µM for compound **46** on *Lm*GDP-MP and compound **100** on *h*GDP-MP, and 0–100 µM for compounds **83** and **92** on *Lm*GDP-MP and compound **100** on *Ld*GDP-MP. Man-1-P was used at 10–100 µM for *Ld*GDP-MP and *h*GDP-MP and 2.5–50 µM for *Lm*GDP-MP. GTP was held constant at 100 µM for *Ld*GDP-MP and *h*GDP-MP, and 50 µM for *Lm*GDP-MP. The formula of each compound is represented on the left of the figure. The compounds *K*_*i*_ are indicated on each plot, as well as the type of inhibition. ND: no *K*_*i*_ could be determined since the double reciprocal plots did not fit with any (competitive, non-competitive, uncompetitive or mixed) inhibition model. The results expressed correspond to the mean of three independent experiments ± SD.

Based on 3D-models of leishmanial GDP-MPs, an in-house library of 100 compounds was designed and synthesized^6, 7, 23^. The activity of these compounds was firstly evaluated at a concentration of 100 µM on the three purified GDP-MPs to select the molecules that specifically inhibit the enzyme of the parasite (Fig. 5a). Among the 100 products evaluated, 11 showed an enzyme inhibition above 50% on a leishmanial GDP-MP, namely compounds **46**, **47**, **71**, **76**, **81**, **82**, **83**, **92**, **98**, **99** and **100**. In order to determine the affinity of these compounds for the GDP-MPs, their *K*_*i*_ was investigated on the three purified enzymes. Out of the 11 molecules, only 5 (**46**, **83**, **92**, **99** and **100**) exhibit a significant *K*_*i*_ on a leishmanial GDP-MP (Fig. 5b), indicating that the 6 remaining products have a low affinity for the enzyme of the parasite. With a promising *K*_*i*_ at 7.00 ± 3.39 µM, compound **99** inhibits specifically and competitively *Ld*GDP-MP, but not the two other purified GDP-MPs, for which no *K*_*i*_ could be determined (Fig. 5b). A competitive inhibition was also observed with compound **100** on both *Ld*GDP-MP and *h*GDP-MP with *K*_*i*_ values at 61.79 ± 16.32 µM and 19.74 ± 3.87 µM, respectively (Fig. 5b). These results show that despite a modest activity on *Ld*GDP-MP, compound **100** could inhibit *h*GDP-MP with a higher affinity. Compounds **46**, **83** and **92** exhibited *K*_*i*_ values between 15 to 25 µM on *Lm*GDP-MP. No *K*_*i*_ was obtained with these products on *Ld*GDP-MP or *h*GDP-MP indicating a specificity of action on *Lm*GDP-MP. Nonetheless, these compounds exert a non-competitive (compounds **46** and **92**) or uncompetitive (compound **83**) inhibition on *Lm*GDP-MP (Fig. 5b). The types of inhibition were also graphically determined and confirmed by Dixon plots for competitive or non-competitive inhibitors or Cornish-Bowden plots for uncompetitive inhibitors (Supplementary Fig. S6).

### Docking analysis of competitive inhibitors

**Figure 6:**
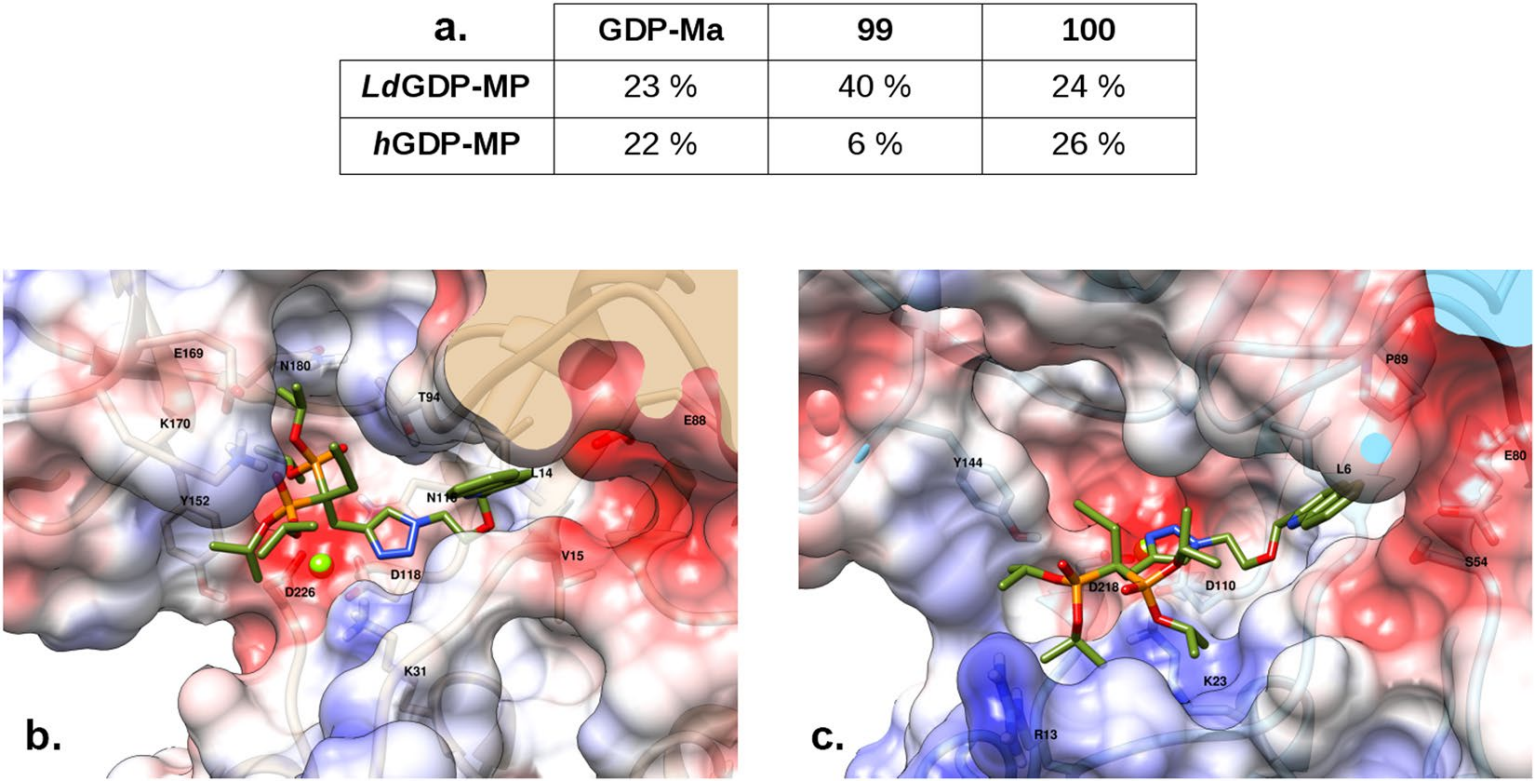
Docking analysis of compounds **99** and **100** on *Ld*GDP-MP and *h*GDP-MP. (**a**) Percentage of “good” poses of GDP-Mannose, compound **99** and **100** over the hundred conformations generated by the ten docking calculations onto *Ld*GDP-MP and *h*GDP-MP. For GDP-Mannose, a “good” pose is the one observed in the PDB crystallographic structure 2X5Z. For the two compounds **99** and **100**, a “good” pose has the quinoline group located on the GDP-Mannose guanine. (**b,c**) Position and orientation of the lowest energy “good” pose of compound **99** in the catalytic site of *Ld*GDP-MP (**b**) and hGDP-MP (**c**). The protein surface is colored as a function of the charge density (red, white and blue colors indicating negative, neutral and positive area, respectively). The amino-acids that make contacts with compound **99** are indicated by their one-letter code and number in the sequence.

In order to further study and compare the interactions of competitive inhibitors identified in this work (compounds **99** and **100**) in the catalytic site of *Ld*GDP-MP and *h*GDP-MP, a docking analysis of these products was performed on structural models of the enzymes^7^, with the natural ligand GDP-mannose used as a reference (Fig. 6a). While compound **100** binds to both *Ld*GDP-MP and *h*GDP-MP with similar potency as for GDP-mannose, compound **99** binds preferentially and more potently to *Ld*GDP-MP. These results are in good agreement with the *K*_*i*_ measurements of these two competitive inhibitors on *Ld*GDP-MP and *h*GDP-MP (Fig. 5b). Furthermore, the preferential position of the quinoline group of compound **99** is similar in both enzyme species, while its diisopropylphosphonate groups are located more deeply in the *Ld*GDP-MP binding site (Fig. 6b) than in the *h*GDP-MP (Fig. 6c), in line with the more potent activity of compound **99** on *Ld*GDP-MP than on *h*GDP-MP. More specifically, the quinoline moiety is surrounded by the three residues L14, V15 and E88 in *Ld*GDP-MP, and, similarly, by the four residues L6, S54, E80 and P89 in *h*GDP-MP. In both enzyme species, the magnesium ion is chelated by two aspartate residues (D188 and D226 in *Ld*GDP-MP, and D110 and D218 in *h*GDP-MP) and makes favorable electrostatic interactions with the triazole nitrogens. Nevertheless, the triazole cycle is more tightly held by the three residues K31, T94 and N116 in *Ld*GDP-MP than by the only residue K23 in *h*GDP-MP. But the most striking difference is that the diisopropylphosphonate groups interact with the residues Y152, E169, K170 and N180 in *Ld*GDP-MP, whereas they only interact with Y144 and R13 (which is located slightly outside of the active site) in *h*GDP-MP. When comparing the pose of compound **99** in the protein catalytic site with the one of GDP-Mannose^7^, it can be noted that one of its two diisopropylphosphonate groups is located in the cavity that is naturally occupied by the mannose group in *Ld*GDP-MP, but this cavity is not filled by compound **99** in *h*GDP-MP. Altogether, these docking results confirm the selective and competitive inhibition observed with compound **99** on *Ld*GDP-MP (Fig. 5b).

### *In vitro* antileishmanial activity and cytotoxicity of compounds 46, 83, 92, 99 and 100

The antileishmanial activity of compounds **46**, **83**, **92**, **99** and **100** was investigated on both axenic and intramacrophage amastigotes of *L. donovani* and *L. mexicana* on two cell host models, RAW264.7 macrophages and bone marrow derived macrophages (BMDM), the latter being closer to clinical conditions (Table 1). Concerning the RAW264.7 model, compound **99** shows a promising IC_50_ on both axenic and intramacrophage amastigotes of *L. donovani* at 1.06 ± 0.10 µM and 0.63 ± 0.14 µM, respectively. However, this compound has a CC_50_ at 1.53 ± 0.17 µM resulting in a modest but noticeable SI value of 2.4 on *L. donovani*. Interestingly, compound **99** did not show any activity on axenic amastigotes of *L. mexicana*, showing thus a specificity of action on *L. donovani* which is in agreement with the enzymatic results (Fig. 5b). Compound **100** showed similar antileishmanial activities of both parasite species with IC_50_ between approximately 30 to 50 µM and 20 to 27.5 µM on axenic and intramacrophage amastigotes, respectively. With a cytotoxicity at 62.06 ± 7.39 µM, the SI of this compound is comparable to compound **99** on both *L. donovani* and *L. mexicana*. The IC_50_ of compounds **46**, **83** and **92** is above 100 µM on axenic amastigotes of *L. donovani*, showing a specific activity on *L. mexicana* which is consistent with the GDP-MP inhibiton assays (Fig. 5b). Compound **46** presents an IC_50_ at 7.69 ± 0.56 µM and 11.72 ± 1.13 µM on axenic and intramacrophage amastigotes of *L. mexicana*, respectively. Interestingly, compound **92** shows an IC_50_ on intramacrophage amastigotes of *L. mexicana* below 10 µM. With an absence of cytotoxicity at 100 µM, compounds **46** and **92** exhibit attractive SI above 8.5 and 12.1 on *L. mexicana*, respectively. Compound **83** exhibits a moderate antileishmanial activity with IC_50_ between 30 and 45 µM on both amastigote forms of *L. mexicana*. On the BMDM model, compound **99** appears to be the most promising one as it is active both on *L. donovani* and *L. mexicana* intramacrophage amastigotes at the 1–10 micromolar range with the best selectivity index. On both host models, compound **99** showed the highest selectivity index, rendering this compound as the most promising one.

**Table 1:** Antileishmanial and cytotoxic activities of compounds 46, 83, 92, 99 and 100.

Compounds	IC_50_ (µM) ± SD								Selectivity index (CC_50_/IC_50_)			
	*L. donovani*			*L. mexicana*			Cytotoxicity CC_50_ (µM) ± SD		*L. donovani*		*L. mexicana*	
	axenic amastigotes	infected RAW.264.7 macrophages	infected BMDM^b^	axenic amastigotes	infected RAW.264.7 macrophages	infected BMDM^b^	RAW264.7 macrophages	BMDM^b^	RAW264.7 macrophages	BMDM^b^	RAW264.7 macrophages	BMDM^b^
**46**	>100	>100	12.54 ± 0.93	7.69 ± 0.56	11.72 ± 1.13	15.71 ± 3.48	>100	13.10 ± 1.67	—	1.0	>8.5	0.8
**83**	>100	51.88 ± 7.49	27.14 ± 2.04	30.64 ± 3.29	45.41 ± 6.21	25.92 ± 5.37	>100	23.76 ± 1.98	>1.9	0.9	>2.2	0.9
**92**	>100	41.60 ± 13.99	82.45 ± 7.61	43.15 ± 3.42	8.25 ± 1.07	79.29 ± 9.76	>100	77.54 ± 5.34	>2.4	0.9	>12.1	1.0
**99**	1.06 ± 0.10	0.63 ± 0.14	1.06 ± 0.41	>100	1.49 ± 0.26	8.59 ± 2.44	1.53 ± 0.17	>100	2.4	>94.3	1.0	>11.6
**100**	30.68 ± 6.62	19.52 ± 4.53	12.18 ± 4.74	49.25 ± 0.26	27.52 ± 2.23	12.05 ± 1.27	62.06 ± 7.39	51.36 ± 3.45	3.2	4.2	2.3	4.3
Miltefosine^a^	2.08 ± 0.24	1.83 ± 0.22	0.83 ± 0.12	23.74 ± 2.81	52.62 ± 4.98	51.39 ± 5.34	>25	>25	13.7	>30.1	>0.5	>0.5

The results expressed correspond to the mean of three independent experiments (±SD).

(a) Miltefosine: reference compound.

(b) BMDM: Bone Marrow Derived Macrophages.

## Discussion

In this work, two leishmanial GDP-MPs from two geographically distant species (*Ld*GDP-MP and *Lm*GDP-MP), as well as the most homologous human counterpart (*h*GDP-MP) were expressed and purified in order to compare their enzymatic properties and to select new specific antileishmanial agents. Following expression of recombinant GDP-MPs, several successive chromatography methods were used for proteins purification. Size exclusion chromatography showed that *Ld*GDP-MP adopts different oligomerization states: one at very high molecular weight presumably corresponding to soluble aggregates, and three others matching with hexamer, trimer and monomer forms of the enzyme. Several approaches were used to dissociate *Ld*GDP-MP soluble aggregates into lower molecular weight oligomeric forms. Co-expression with chaperone proteins, changing buffer conditions (pH, salt concentration, glycerol) or different types of chromatography were unfruitful. An attempt to fuse *Ld*GDP-MP with MBP tag was also performed, but without success. Nonetheless, according to DLS analysis, the concentrated pool of fractions of peaks 2, 3 and 4 is predominantly in the hexamer form and could be used for enzyme assays, showing that *Ld*GDP-MP has the propensity to hexamerize when the concentration of enzyme is increased. Similar phenomena have been observed for *Lm*GDP-MP, whose hexamerization was dependent on enzyme concentration^21^. Moreover, *Lm*GDP-MP gel filtration profile showed a single peak corresponding to a hexamer, which is in agreement to our study (Fig. 1e). These results indicate that the *Lm*GDP-MP quaternary structure would be more stable than *Ld*GDP-MP. Although *Lm*GDP-MP and *Ld*GDP-MP share 94% identity and amino acids directly involved in substrate binding are conserved in both enzymes, the difference observed in catalytic efficiency between both GDP-MPs (Fig. 3g) could be explained by residues that would influence protein quaternary structure and thus enzyme activity. Indeed, in other NTP transferases, such as UDP-glucose pyrophosphorylase, single amino acid replacements have been reported to alter the quaternary structure as well as the activity of the enzyme^24^. Furthermore, the hexameric form of *Lm*GDP-MP has been shown to dissociate in trimers and monomers at low ionic strength^21^. Thus, once diluted in low ionic strength conditions for enzymatic assays, both *Lm*GDP-MP and *Ld*GDP-MP hexamers could dissociate in a mixture of oligomeric forms that would be able to catalyze the enzyme reaction. In gel filtration, *h*GDP-MP is eluted as a dimer showing that this subunit is able to self-associate. In mammalian, native GDP-MP is an oligomer of about 450 kDa composed of subunits α and β, confirming a self-association of both subunits to form a high molecular weight complex^18, 25^. This native complex has been described to synthesize both GDP-mannose and GDP-glucose. However, only the β subunit has a high activity in synthesizing GDP-mannose. Likewise, in our study, no significant activity was observed with *h*GDP-MP in the presence of substrate analogues such as Glu-1-P sugar monophosphate, or nucleotide triphosphates. Only ITP exhibited 24% of activity on *h*GDP-MP compared to GTP. Similar results were obtained with leishmanial GDP-MPs, showing that the three purified enzymes present high substrate specificity. These data are consistent with the high substrate specificity described in the *T. brucei* GDP-MP, except that this enzyme is also able to use ATP as a substrate with a high *K*_*m*_ at 290 µM^15^. In addition, a high cofactor specificity was observed with the three purified enzymes, except that *Ld*GDP-MP is also able to generate a moderate activity in the presence of Mn^2+^. Surprisingly, *h*GDP-MP has been previously reported to be active in the presence of Mn^2+^, but at a concentration 10 times lower than in our conditions^18^.

Our enzymatic assays showed that the three GDP-MPs have very similar optimal reaction conditions with the concentration of Mg^2+^ ranging from 1 mM to 5 mM and a slightly more acidic pH for *h*GDP-MP compared to the leishmanial enzymes. Except for *Lm*GDP-MP which presents a *K*_*m*_ at 7.67 ± 2.28 µM for GTP, a moderately higher affinity was observed with *h*GDP-MP for both substrates (*K*_*m*_ ≈ 7 µM) compared to *Ld*GDP-MP and *Lm*GDP-MP (*K*_*m*_ ≈ 10–17 µM). These micromolar *K*_*m*_ values are consistent with those obtained with bacterial or trypanosomal GDP-MPs^14, 15^. The investigation of substrate binding order showed that the three purified GDP-MPs exhibit a sequential random mechanism. Although several NTP transferases, such a bacterial GDP-MP or diverse ADP-glucose or TDP-glucose pyrophosphorylases, have been described to exhibit a sequential ordered mechanism, with the NTP binding prior to sugar-1-P^14, 26–28^, a sequential random mechanism was observed in a mammalian UDP-glucose pyrophosphorylase^29^ which is consistent with our findings on human and leishmanial GDP-MPs.

From an in-house library of 100 compounds designed and synthesized from molecular models of leishmanial GDP-MPs^6, 7, 23^, 11 molecules presented *Ld*GDP-MP and/or *Lm*GDP-MP inhibition above 50%, and 5 exhibit a *K*_*i*_ on a leishmanial GDP-MP at the micromolar range. Among these 5 compounds, three are derivatives of quinoline substituted in position 2 (compounds **99** and **92**) or 4 (compound **100**). Compound **100**, a 4-substituted quinoline with a chloride in position 7, has been previously described to be the most potent *L. major* GDP-MP inhibitor identified by high throughput screening with an IC_50_ at 0.58 µM^8^. This compound presents an IC_50_ on *L. donovani* and *L. mexicana* intramacrophage amastigotes between 20 and 30 µM, which is consistent with the IC_50_ previously reported at 21.9 µM in *L. major* ^8^. Nonetheless, compound **100** exhibits only moderate or no inhibition on the leishmanial enzymes *Ld*GDP-MP and *Lm*GDP-MP, respectively, while *h*GDP-MP is inhibited with a noticeable *K*_*i*_ at around 20 µM. Thus, this inhibitor could probably not be used against *L. donovani* or *L. mexicana* for the development of new antileishmanials. Indeed, 2-substituted quinolines have been previously shown to present promising antileishmanial activities both *in vitro* and *in vivo* ^11, 30^. Thus, the presence of a 2-substituted quinoline in leishmanial GDP-MP inhibitors could potentiate their antileishmanial activities through GDP-MP inhibition. The most active and specific competitive inhibitor on *Ld*GDP-MP is the compound **99** (*K*_*i*_ of 7 µM) which does not inhibit the other GDP-MPs studied. This result is in agreement with the docking analyses showing a significant higher potency of binding of the compound **99** in the catalytic pocket of *Ld*GDP-MP compared to *h*GDP-MP. Indeed, this 2-substituted quinoline presents a diisopropylphosphonate group located in place of the mannose group of GDP-mannose which could account for the competitive inhibition activity of the molecule. Compound **99** has been designed from a structural analysis of GDP-MP active site molecular models with the aim to get inhibitors with molecular volumes suitable for filling the catalytic pocket. Consequently, this compound initially designed as a substrate analogue has a chemical structure with substituants able to achieve these criteria. Regarding the *K*_*i*_ values of all evaluated inhibitors, compound **99** is the inhibitor presenting the highest affinity for a leishmanial GDP-MP. Although compound **99** exhibits a very interesting IC_50_ on intramacrophage parasites at the sub-micromolar range and is specifically active on axenic amastigotes of *L. donovani*, it presents a substantial cytotoxicity leading to a SI value of 2.4 on the RAW264.7 model. However, in the BMDM model, it is active both on *L. donovani* and *L. mexicana* intramacrophage amastigotes without any cytotoxicity. These discrepancies can be ascribed to different mechanisms of uptake and accumulation within the host cell. Nevertheless, both BMDM and RAW264.7 cell models are recognized and routinely used in classical drug screening and to decipher cell mechanisms in *Leishmania* ^31–35^. It is not surprising to observe discrepancies from one model to another as a function of an evaluated chemical series. In our case, the most promising inhibitor, compound **99**, exhibited this tendency with CC_50_ values at 1.53 µM and >100 µM on RAW264.7 and BMDM cell models, respectively. However, it is the most active compound on both *L. donovani* and *L. mexicana* intramacrophage amastigotes. These promising results have now to be confirmed by a further *in vivo* evaluation. Moreover, in a subsequent fundamental study focused on GDP-MP cell biology, we will extend the investigation by comparing the results in diverse cell models, including human ones, in order to understand the mechanism of action of compound **99**. Additionally, this compound could be further used as a pharmacological lead compound to develop new antileishmanial drugs that would be specifically active on the parasite, without toxicity on mammalian host. Compound **92**, which is also a 2-substituted quinoline with a chloride in position 6 and a diphenyl group in place of the 2 diisopropylphosphonates of compound **99**, exhibits a specific inhibition on *Lm*GDP-MP, and not on the other GDP-MPs studied. This specific inhibition of *Lm*GDP-MP is also observed with compounds **46** and **83**, which are quinoxaline and guanine derivatives, respectively. Interestingly, no activity was observed on *L. donovani* axenic amastigotes with compounds **46**, **83** and **92**, which is consistent with the specific activity observed on *Lm*GDP-MP (Fig. 5b). Interestingly, these three inhibitors do not present any cytotoxicity on RAW264.7 macrophages, leading to an attractive SI above 8.5 and 12.5 for compounds **46** and **92**, respectively. Compound **83** exhibits an uncompetitive inhibition on *Lm*GDP-MP and thus binds only to the complex formed between the enzyme and the substrate, while compounds **46** and **92** present a non-competitive inhibition on the same leishmanial enzyme and can bind to the enzyme indifferently of the presence of the substrate. These results indicate that these three compounds are potential allosteric inhibitors of *Lm*GDP-MP. Indeed, targeting allosteric sites has been previously shown to provide new opportunities for the development of specific enzyme inhibitors^36^.

Altogether, these results allowed identifying compounds **46**, **83**, **92** and **99** as pharmacological tools which could be further pharmacomodulated to improve their specificity and affinity for leishmanial GDP-MPs as well as their antileishmanial activities, and to generate new specific antileishmanial drugs and expand the current chemotherapeutic arsenal for the treatment of leishmaniasis. This study also shows that a conservation of amino acids at the level of the catalytic site does not prevent to get specific inhibitors since probable extra-catalytic site interactions could occur between the inhibitor and the target, as probably happening with compounds **46**, **83** and **92**. More interestingly, compound **99** clearly appears to be the most promising one for the following reasons: (i) it is the single competitive inhibitor of *Ld*GDP-MP, (ii) it is active both on *L. donovani* and *L. mexicana* intramacrophage amastigotes at the micromolar range in the BMDM model, (iii) its selectivity index values are higher than 10 on the BMDM host cell model. As perspectives, compound **99** is worth of further *in vivo* evaluation on *L. donovani* Balb/c mice model. In addition, next pharmacomodulations will be set up to understand the structure-activity relationship of derivatives of this lead compound towards GDP-MP and intracellular parasites through deciphering their uptake mechanism.

## Methods

### Chemical synthesis

Detailed description of synthetic methods and compounds characterization are provided in the Supplementary Methods. For biological evaluations, all compounds were dissolved in DMSO at 10 mM stock solution, aliquoted, and stored at −80 °C before use.

### Cloning, production and purification of recombinants GDP-MPs

Detailed materials and methods of cloning, production and purification of *Ld*GDP-MP, *Lm*GDP-MP and *h*GDP-MP are provided in Supplementary Methods. The band stained by Coomassie blue on SDS-PAGE corresponding to the each purified *Ld*GDP-MP, *Lm*GDP-MP or *h*GDP-MP was excised, digested and analyzed by peptide mass fingerprint using MALDI-TOF mass spectrometry as previously described^37^.

### Enzymatic assays

GDP-MP catalyzes the following reaction:

$${\rm{Man}}\, \mbox{-} \,1\, \mbox{-} \,{\rm{P}}+{\rm{GTP}}\to {\rm{GDP}}-{\rm{Mannose}}+{\rm{Pyrophosphate}}$$

The reaction media were buffered at pH optima and contained optimal concentrations of Man-1-P, GTP, GDP-MP and Mg^2+^ determined for each purified GDP-MP (Fig. 2d), supplemented with DTT (1 mM). Inorganic pyrophosphatase, which hydrolyses the inorganic pyrophosphates generated by the GDP-MPs in inorganic phosphates, was added at 0.1 U/ml (Sigma-Aldrich). Inorganic pyrophosphatase was added in excess to the reaction medium in order to minimize the probability that the putative inhibitors of GDP-MPs interfere with this enzyme. The reaction was initiated by the addition of GDP-MP, and was stopped after adding 100 µl of revelation buffer containing malachite green (0.03% w/v), ammonium molybdate (0.2% w/v) and Triton X-100 (0.05% v/v) in HCl (0.7 M) for 5 min at the optimal temperature^38^. The quantity of inorganic phosphate generated, representing GDP-MP activity, was determined by measuring OD_650nm_ (Lab systems Multiskan MS). The molar extinction coefficient ε of the end product detected was determined by measuring OD_650nm_ with a range of inorganic phosphate concentrations diluted in the revelation buffer and was calculated at 27207 M^−1^ cm^−1^ (Supplementary Fig. S2). The kinetic constants *K*_*m*_, *V*_*m*_ and *k*_*cat*_ were determined by non linear regression using GraphPad Prism 6.0.

### Evaluation of compounds on purified GDP-MPs

Enzyme activity was measured as described above in the presence of 100 µM of compounds and the percentage of inhibition was calculated in reference to untreated enzyme. As a control, compounds were evaluated in parallel in the same conditions on inorganic pyrophosphatase alone in order to discard false-positives. No significant inhibition (<5%) of inorganic pyrophosphatase was observed with the positive compounds (**46**, **83**, **92**, **99** and **100**) selected in this study. Furthermore, no effect was observed on the activity of the three purified GDP-MPs with 1% DMSO, corresponding to the maximal solvent concentration used at 100 µM of compound. Compounds presenting a minimum of 50% inhibition on a leishmanial GDP-MP were subsequently selected to determine their type of inhibition and their *K*_*i*_ values. Inhibition data were fitted to competitive, non-competitive or uncompetitive models of enzyme inhibition by non linear least squares regression analysis using GraphPad Prism 6.0.

### Cell Cultures

All details of bacterial, parasite, macrophage cultures are described in Supplementary Methods.

### *In vitro* antileishmanial evaluation of compounds on axenic and intramacrophage amastigotes

The evaluations of activity on axenic and intramacrophage amastigotes were adapted from the protocols previously described^39^. Briefly, for the evaluation on axenic amastigotes, two fold serial dilutions of the compounds from a maximal concentration of 100 µM were performed in 100 µl of complete medium (see above) in 96-well microplates. Axenic amastigotes were then added to each well at a density of 10^6^/ml in a 200 µl final volume. After 72 h of treatment at 37 °C for *L. donovani* or at 32 °C for *L. mexicana* with 5% CO_2_, 20 µl of resazurin (450 µM) was added to each well and further incubated in the dark for 24 h at 37 °C for *L. donovani* or at 32 °C for *L. mexicana* with 5% CO_2_. In living cells, resazurin is reduced in resorufin and this conversion is monitored by measuring OD_570nm_ (resorufin) and OD_600nm_ (resazurin; Lab systems Multiskan MS). The activity of the compounds was expressed as IC_50_. Miltefosine was used as the reference drug.

Concerning the evaluation on intramacrophage amastigotes, RAW 264.7 macrophages (from ATCC) and bone marrow derived macrophages (BMDM) were plated in 96-well microplates at a density of 2 × 10^4^ cells per well and incubated for 24 h at 37 °C with 5% CO_2_ for RAW264.7 cells or at 37 °C with 7% CO_2_ for BMDM. Axenic amastigotes were differenciated as described above, centrifuged at 2,000 g for 10 min, resuspended in DMEM complete medium, and added to each well to reach a 16:1 parasite to macrophage ratio. After 24 h of infection at 37 °C with 5% CO_2_ for RAW264.7 cells or at 37 °C with 7% CO_2_ for BMDM, extracellular parasites were removed, and DMEM complete medium (100 µl) containing two fold serial dilutions of the compounds from a maximal concentration of 100 µM was added to each well. After 48 h of treatment, the medium was removed and replaced by DirectPCR Lysis Reagent (100 µl; Euromedex) before 3 freeze-thaw cycles at room temperature, addition of 50 µg/ml proteinase K, and a final incubation at 55 °C overnight to allow cell lysis. 10 µl of each cell extract was then added to 40 µl of DirectPCR Lysis reagent containing Sybr Green I (0.05%; Invitrogen). DNA fluorescence was monitored using Mastercycler^®^ realplex (Eppendorf). The activity of the compounds was expressed as IC_50_. Miltefosine was used as the reference drug.

### Evaluation of compounds cytotoxicity

Cytotoxicity was evaluated on RAW 264.7 macrophages and BMDM. Cells were plated in 96-well microplates at a density of 2 × 10^4^ cells per well. After an incubation of 24 h at 37 °C with 5% CO_2_ for RAW264.7 cells or at 37 °C with 7% CO_2_ for BMDM, the medium was removed in each well, and 100 µl of DMEM complete medium containing two fold serial dilutions of the compounds was added to each well. After 48 h of incubation at 37 °C with 5% CO_2_ for RAW264.7 cells or at 37 °C with 7% CO_2_ for BMDM, 10 µl of resazurin (450 µM) was added to each well, and further incubated in the dark for 4 h at 37 °C with 5% CO_2_. Cell viability was then monitored as described above. The cytotoxicity of the compounds was expressed as CC_50_ (Cytotoxic Concentration 50%: concentration inhibiting the macrophages growth by 50%).

### Molecular docking

To provide a structural basis for the activity of competitive inhibitors on GDP-MPs, three-dimensional structures of enzyme-ligand complexes were predicted using docking calculations. All details of docking calculations are described in Supplementary Methods.

## Electronic supplementary material

Supplementary information
