# Supplementary material for: Biochemical analysis of leishmanial and human GDP-Mannose Pyrophosphorylases and selection of inhibitors as new leads
Source: Sci Rep. 2017 Apr 7;7:751. doi: 10.1038/s41598-017-00848-8 (PMC5429698; doi:10.1038/s41598-017-00848-8)
Supplement: Supplementary file 1 — Supplementary information [file 41598_2017_848_MOESM1_ESM.pdf]

Biochemical analysis of leishmanial and human GDP-mannose Pyrophosphorylases and selection of inhibitors as new leads

Wei Mao[1], Pierre Daligaux[1], Noureddine Lazar[2], Tâp Ha-Duong[3], Christian Cavé[1], Herman van Tilbeurgh[2], Philippe M. Loiseau[1], Sébastien Pomel[1*]

[1]Chimiothérapie antiparasitaire, UMR 8076 CNRS BioCIS, Université Paris Sud, Université Paris-Saclay, 92296, Châtenay-Malabry, France
[2]Institut de Biologie Intégrative de la Cellule, UMR 9198, CNRS, Université Paris Sud, Université Paris-Saclay, Bâtiment 430, 91405, Orsay, France
[3]Molécules Fluorées et Chimie Médicinale, UMR 8076 CNRS BioCIS, Université Paris Sud, Université Paris-Saclay, 5 rue Jean-Baptiste Clément, 92296, Châtenay-Malabry, France
[*]Correspondence: sebastien.pomel@u-psud.fr

Supplementary Table 1: Dynamic Light Scattering (DLS) analysis of *Ld*GDP-MP

	R^a (nm)	Polydispersity[b] (%)	MW-R^c (kDa)	Intensity[d] (%)
Peak 1	16.5	44.3	2373	67.3
Peaks 2-4	6.2	14.3	237	37.7

(a) Hydrodynamic radius
(b) Percentage of polydispersity
(c) Molecular weight estimated via the hydrodynamic radius $R=1.29*3*(M/110)^{1/3}$
(d) Percentage of intensity of the scattered light

DLS experiments were performed by using DynaPro™ Titan (Wyatt Technology)

Supplementary Figure 1. Mass spectrometry analysis of the purified GDP-MPs.
(a-c) MALDI-TOF peptide mass fingerprints of the purified *Ld*GDP-MP (a), *Lm*GDP-MP (b) and *h*GDP-MP (c). The peptides identified are highlighted in red. The percentage of sequence coverage is mentioned above each sequence.

a.

*Ld*GDP-MP : protein sequence coverage: 63%

```
  1 MSASDGQGMR AVILVGGFGT RLRPLTLTTP KPLVPFCNKP MIIHQIEALK
 51 AVGVTEVILA VAYRPEAMKE QMDEWSRKLG VSFVFSVEEE PLGTAGPLAL
101 ARDILMQDDK PFFVLNSDVT CTFPMQELLD FHKAHGGEGT IMVSQVTQWE
151 KYGVVVYSPQ NYQIERFVEK PSRFLGDRIN AGIYIFNKSI LDRIPPRRTS
201 IEKEIFPAMA AEGQLYAFNL EGFWMDVGQP KDYILGMTKF IPSLVHGNRE
251 TEQLHTEAME HQRGGRFTVI GASLIDPSAK IGDGAVIGPY ASIGANCVIG
301 ESCRIDNAAI LENSKVGKGT MVSRSIVGWN NRIGSWCHIK DISVLGDDVE
351 VKDGVILIGT KVLPNKDVGE HRFEPGIIM
```

b.

*Lm*GDP-MP : protein sequence coverage: 41%

```
  1 MSASDGQGMR AVILVGGFGT RLRPLTLTTP KPLVPFCNKP MIIHQIEALK
 51 AVGVTEVILA VAYRPEAMKE QMDEWSRKLG VSFVFSVEED PLGTAGPLAL
101 ARDILMQDDK PFFVLNSDVT CMFPLQELLD FHKARGGEGT IMVSQVTQWE
151 KYGVVVYSQQ SYQIERFVEK PSSFLGDRVN AGIYIFNKSI LDRIPPCRTS
201 IEKEIFPAMA AEGELYAFNL EGFWMDVGQP KDYILGMTKF IPSLLDGDRK
251 TEQLHTEATE HQHGGRFTVV GASLIDPSAK IGDGAVIGPC ASIGANCVIG
301 ESCRIDNAAI LENSKVGKGT MVSRSIVGWN NRIGSWCHIE DISVLGDDVE
351 VKDGVVLIGT KVLPNKDVGE HHFQAGIIM
```

c.

*h*GDP-MP : protein sequence coverage: 63%

```
  1 MKALILVGGY GTRLRPLTLS TPKPLVDFCN KPILLHQVEA LAAAGVDHVI
 51 LAVSYMSQVL EKEMKAQEQR LGIRISMSHE EEPLGTAGPL ALARDLLSET
101 ADPFFVLNSD VICDFPFQAM VQFHRHHGQE GSILVTKVEE PSKYGVVVCE
151 ADTGRIHRFV EKPQVFVSNK INAGMYILSP AVLQRIQLQP TSIEKEVFPI
201 MAKEGQLYAM ELQGFWMDIG QPKDFLTGMC LFLQSLRQKQ PERLCSGPGI
251 VGNVLVDPSA RIGQNCSIGP NVSLGPGVVV EDGVCIRRCT VLRDARIRSH
301 SWLESCIVGW RCRVGQWVRM ENVTVLGEDV IVNDELYLNG ASVLPHKSIG
351 ESVPEPRIIM
```

Supplementary Figure 2. Determination of the molar extinction coefficient (ε) of the reaction

The OD_{650nm} is plotted as a function of inorganic phosphate (Pi) concentration. The ε is the slope of the curve and is calculated at 27207 M^{-1} cm^{-1}. The results expressed correspond to the mean of three independent experiments ± SD.

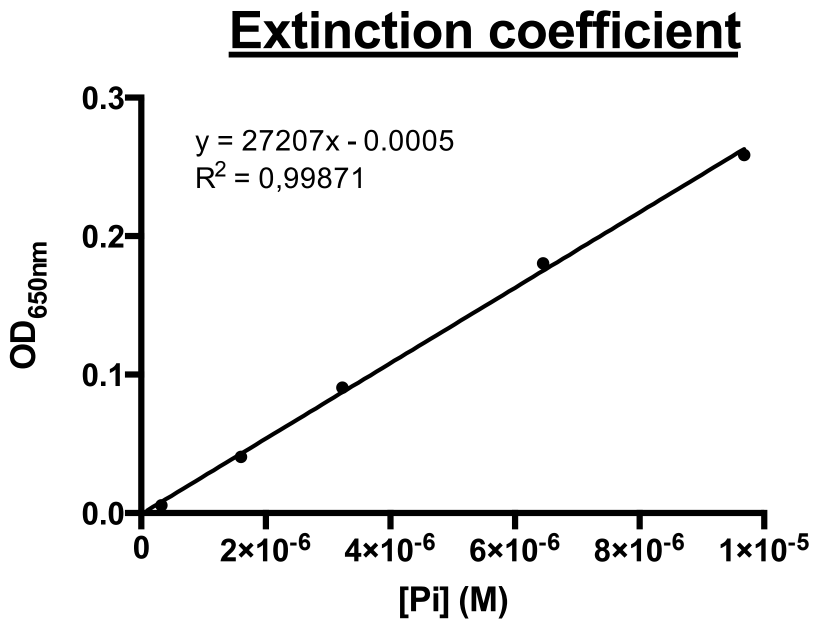

Supplementary Figure 3. Determination of optimal reaction time of *Ld*GDP-MP, *Lm*GDP-MP and *h*GDP-MP.

The OD_{650nm} is plotted as a function of the time of reaction. All reactions were performed at optimal temperature, pH, Mg^{2+}, enzyme and substrates concentrations for each GDP-MP. The results expressed correspond to the mean of three independent experiments.

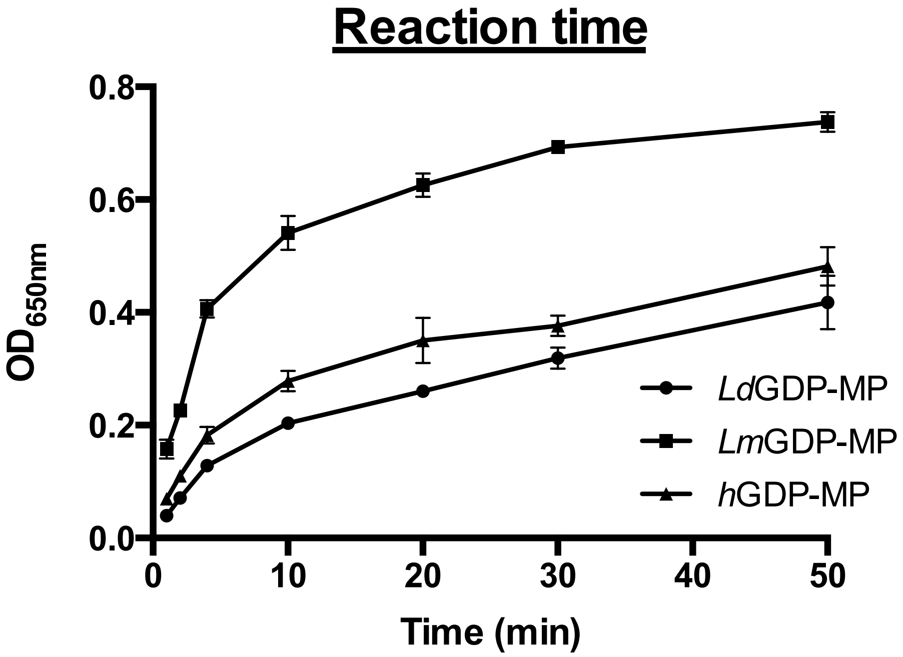

Supplementary Figure 4. Determination of *Ld*GDP-MP, *Lm*GDP-MP and *h*GDP-MP optimal concentrations for enzyme assays.

The OD_{650nm} is plotted as a function of enzymes concentrations. All reactions were performed at optimal time, temperature, pH, Mg^{2+} and substrates concentrations of each GDP-MP. The results expressed correspond to the mean of three independent experiments.

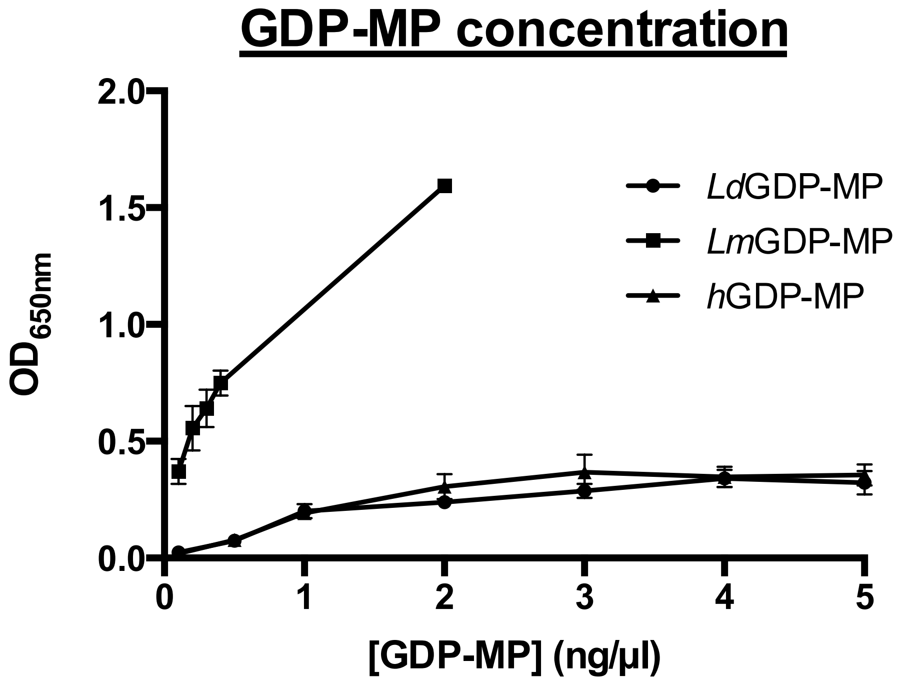

Supplementary Figure 5. Determination of optimal substrate concentrations of *Ld*GDP-MP, *Lm*GDP-MP and *h*GDP-MP for enzyme assays.

(a-c) Michaelis-Menten plots V = f(/[Man-1-P]) for *Ld*GDP-MP (a), *Lm*GDP-MP (b) and *h*GDP-MP (c) reaction. GTP concentration was held constant at 150 µM for *Ld*GDP-MP and 80 µM for *Lm*GDP-MP and *h*GDP-MP. All reactions were performed at optimal time, temperature, pH, Mg^{2+} and enzyme concentrations of each GDP-MP. The results expressed correspond to the mean of three independent experiments ± SD.

(d-f) Michaelis-Menten plots V = f(/[GTP]) for *Ld*GDP-MP (d), *Lm*GDP-MP (e) and *h*GDP-MP (f) reaction. Man-1-P concentration was held constant at 150 µM for *Ld*GDP-MP and 80 µM for *Lm*GDP-MP and *h*GDP-MP. All reactions were performed at optimal time, temperature, pH, Mg^{2+} and enzyme concentrations of each GDP-MP. The results expressed correspond to the mean of three independent experiments ± SD.

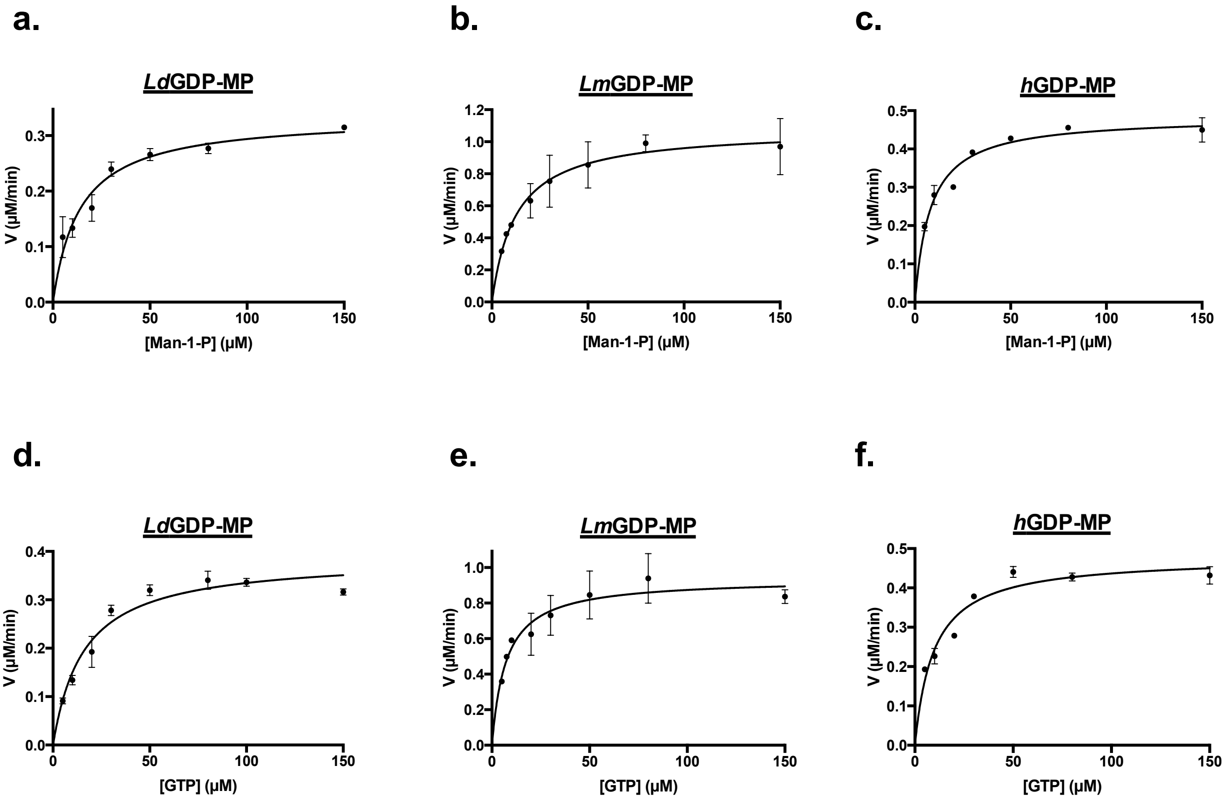

Supplementary Figure 6. Evaluation of activities of compounds 46, 83, 92, 99 and 100 on *Ld*GDP-MP, *Lm*GDP-MP and *h*GDP-MP.
(a-e) Dixon plots 1/V=f([I]) of compounds **99** (a), **100** (b, c), **46** (d) and **92** (e) on *Ld*GDP-MP (a, b), *h*GDP-MP (c) and *Lm*GDP-MP (d, e). The type of inhibition of each compound is indicated on each plot. The results expressed correspond to the mean of three independent experiments ± SD.
(f) Cornish-Bowden plots [Man-1-P]/V=f([I]) of compounds **83** on *Lm*GDP-MP. The type of inhibition is indicated on the plot. The results expressed correspond to the mean of three independent experiments ± SD.

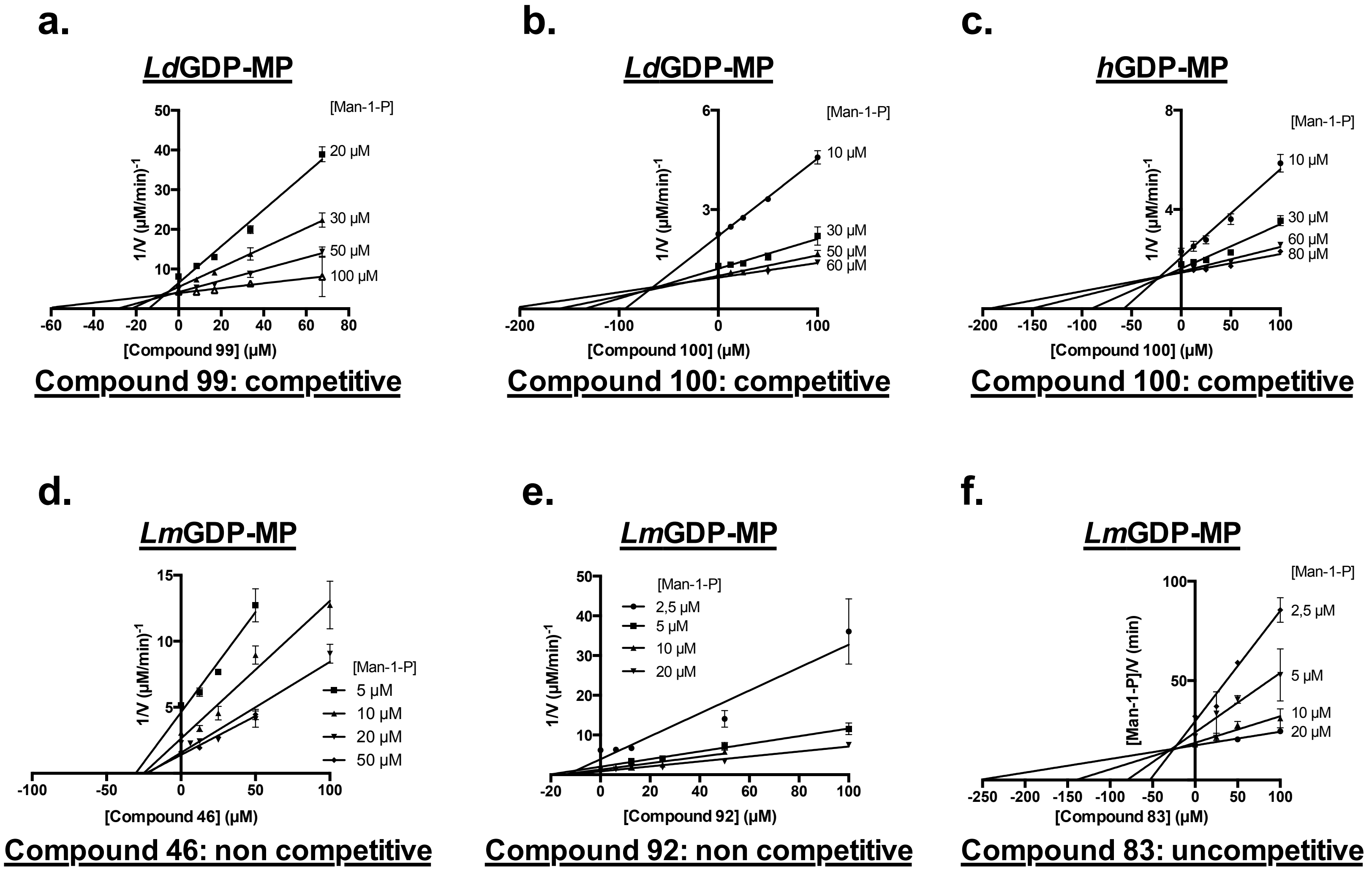

Supplementary Figure 7. Purification of of *Ld*GDP-MP, *Lm*GDP-MP and *h*GDP-MP

(a, c, f) Full length coomassie blue stained SDS-PAGE of *Ld*GDP-MP (a), *Lm*GDP-MP (c), and *h*GDP-MP (f) purified from Ni-NTA column. Lane M: molecular weight marker, Lane TOT: total, Lane SN: supernatant, Lane P: Pellet, Lane FT: flow through, Lane W: wash, Lanes E1, E2 and E4: elution fraction at 100 µM, 200 µM and 400 µM imidazole, respectively. The arrowheads show the proteins of interest. The molecular weights are indicated in kDa.

(b, d, g) Full length coomassie blue stained SDS-PAGE of fraction volumes of peaks 1 (49-51 mL), 2 (66-68 mL), 3 (75-78 mL) and 4 (84-86 mL) of *Ld*GDP-MP (b), peaks 1 (12-13 mL) and 2 (16 mL) of *Lm*GDP-MP (d) and peak 1 (77-82 mL) of *h*GDP-MP (g). Lane M: molecular weight marker, Lane Lo: loading control. The arrowheads show the proteins of interest. The molecular weights are indicated in kDa.

(e) Full length coomassie blue stained SDS-PAGE of a concentrated pool of fractions containing *Lm*GDP-MP eluted from anion exchange chromatography (AEC). Lane M: molecular weight marker. The arrowhead shows the protein of interest. The molecular weights are indicated in kDa.

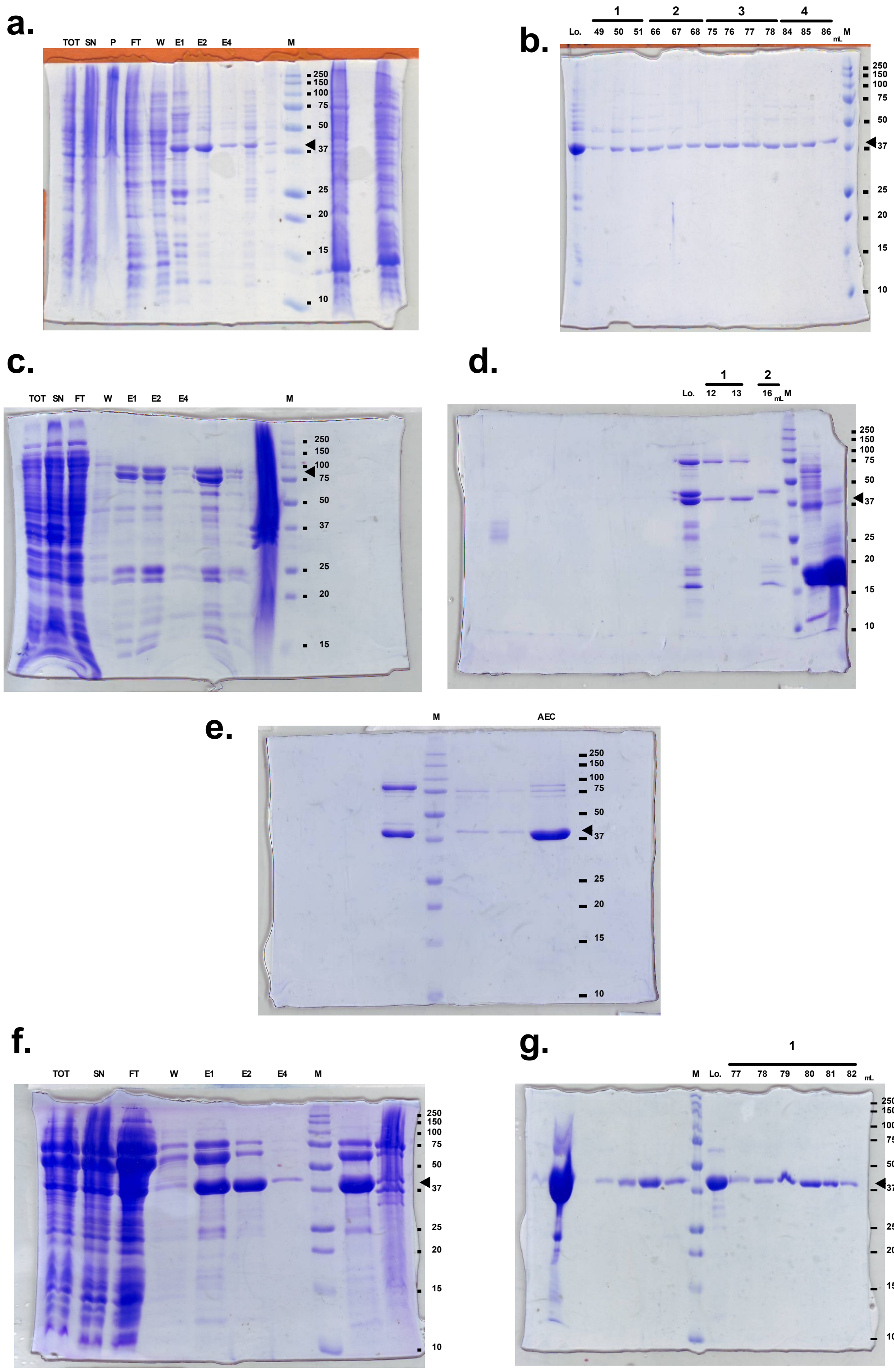

Supplementary Methods

Chemical synthesis

All chemicals and solvents were purchased from Sigma-Aldrich. Organic solvents were dried and purified by standard procedures. The final products were purified with Delta Prep HPLC system (Waters) using a C18 column with UV detection at 250, 260, 280, and 300 nm. The analyses were carried out by HPLC on Sunfire (19×150 mm, particle size 5μm, C18) column. Mass spectra were recorded using a Micromass/Waters LCT ESI-TOF mass spectrometer. TLC was carried out on Kieselgel 60 F254 plates (Merck) in the proper solvent systems (see below) and spots were visualized by UV irradiation, para-anisaldehyde/aqueous sulfuric acid solution. Evaporations were performed under reduced pressure. The column chromatography was performed using silica gel 60 19–37 μm, S.A. 500-600 m^2/g (Alfa Aesar). Eluent composition is given in v/v per cent. NMR spectra were acquired on Bruker AM-300 and AV-200 instruments (Bruker) in appropriate deuterated solvents. Chemical shifts (δ) are reported in ppm relative to the TMS signal. Coupling constants J are reported in Hertz.

From the evaluation on GDP-MPs and on parasites of an in-house library of 100 compounds designed and synthesized from molecular models of leishmanial and human GDP-MPs[1-3], the most promising activities were observed with compounds **46**, **83**, **92**, **99** and **100**. Compounds **83** and **100** were prepared by following the previous literature reports[3,4]. For compounds **46**, **92**, and **99** the last synthesis steps are described below.

<u>Synthesis of compound N-benzyl-2,3-diphenylquinoxaline-6-carboxamide (compound **46**)</u>

This compound was prepared at room temperature by coupling reaction between 2.3-diphenylquinoxaline carboxylic acid and the benzylamine in the presence of propylphosphonic anhydride. The N-benzyl-2,3-diphenylquinoxaline-6-carboxamide was isolated with 45 % yield as a white powder.

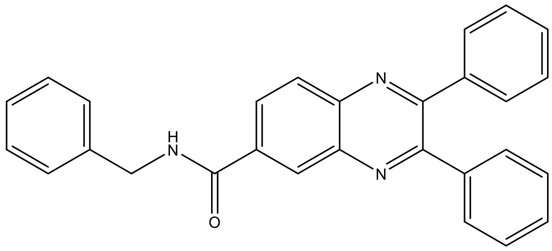

46

^{1}H NMR (300 MHz, CDCl3) d 8.50 (d, J = 1.6 Hz, 1H), 8.21 (dd, J = 8.7, 1.8 Hz, 1H), 8.16 (d, J = 8.7 Hz, 1H), 7.56 – 7.47 (m, 4H), 7.44 – 7.26 (m, 11H), 7.04 (t, J = 5.4 Hz, 1H), 4.69 (d, J = 5.6 Hz, 2H). ^{13}C NMR (75 MHz, CDCl3) d 166.54, 154.74, 154.34, 142.39, 140.35, 138.60, 137.88, 135.61, 129.88, 129.79, 129.68, 129.24, 129.14, 128.84, 128.56, 128.33, 127.94, 127.73, 127.68, 44.41.

<u>Synthesis of 2-((2-(4-([1,1’-biphényl]-4-yl)-1H-1,2,3-triazol-1-yl)éthoxy)méthyl)-6-chloroquinoline (compound **92**)</u>

This compound was prepared by using the copper-catalyzed Huisgen 1,3-dipolar cycloaddition of azides and terminal alkynes that we have previously developed[3]. A solution of corresponding azidoquinoline (1 Eq) and the alkyne (2 Eq) in a mixture of acetonitrile /water (1/1) was treated with $CuSO_4$ (0.25 Eq). Hydrazine hydrate (2 Eq) was added dropwise. at room temperature for 2 days. The crude product was purified by column chromatography (Ethyl acetate/cyclohexane 4/1) to afford **92** as a yellow powder with 73 % yield.

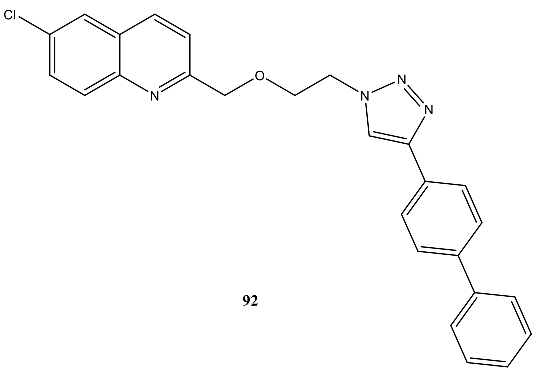

92

^{1}H NMR (300 MHz, $CDCl_3$)δ 8.06 (d, J = 8.5 Hz, 1H), 8.00 (s, 1H), 8.00 (d, J = 8.9 Hz, 1H), 7.91(d, J = 8.4 Hz, 2H), 7.79 (d, J = 2.3 Hz, 1H), 7.67 (dd, J = 12.9, 5.2 Hz, 5H), 7.48 – 7.35 (m, 4H),4.84 (s, 2H), 4.71 (t, 2H), 4.07 (t, 2H). ^{13}C NMR (75 MHz, $CDCl_3$)δ 158.3, 147.5, 145.9, 140.8, 140.5, 136.1, 132.3, 130.7, 130.6, 129.6,128.8, 128.1, 127.5, 126.9, 126.3, 126.0, 120.8, 120.2, 74.5, 69.2, 50.4.HRMS : C26H21ClN4O (M+H)+: calc 441.1482, found 441.1483.

Synthesis of tetraisopropyl (1-(1-(2-(quinolin-2-ylmethoxy)ethyl)-1H-1,2,3-triazol-4-yl)but-3-yne-1,1-diyl)bis(phosphonate) (compound **99**)

This compound was obtained by alkylation reaction of a solution in anhydrous THF of the corresponding bisphosphonate with propargyl bromide in the presence of sodium hydride at 0° C for one day. The reaction mixture was filtered and the filtrate concentrated and the residue purified by column chromatography (toluene/acetone 6/1) to afford **99** as an oil with 62 % yield.

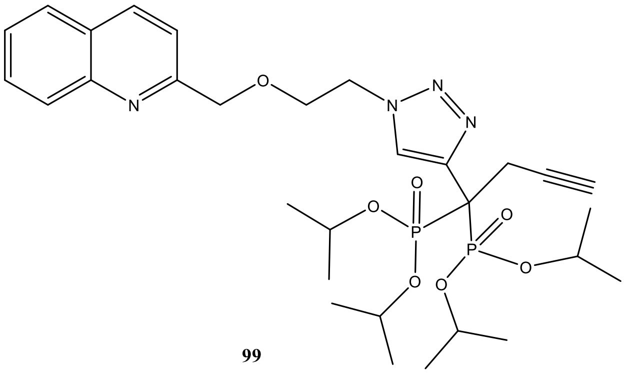

99

^{1}H NMR (300 MHz, CDCl) δ 8.04 (d, J = 8.5 Hz, 1H), 7.95 (d, J = 8.5 Hz, 1H), 7.71 (d, J = 8.1 Hz, 1H), 7.63 (ddd, J = 8.4, 6.9, 1.4 Hz, 1H), 7.54 (s, 1H), 7.48 – 7.43 (m, 1H), 7.40 (d, J = 8.5 Hz, 1H), 6.90 (dt, J = 15.8, 5.8 Hz, 1H), 6.80 (d, J = 15.9 Hz, 1H), 5.13 (d, J = 5.6 Hz, 2H), 4.76 – 4.55 (m, 4H), 3.45 (t, J = 5.4 Hz, 2H), 2.78 (td, J = 14.9, 2.7 Hz, 2H), 1.94 (t, J = 2.7 Hz, 1H), 1.27 – 1.16 (m, 24H). ^{13}C NMR (75MHz, CDCl) δ 154.1, 148.0, 146.0, 145.9, 145.8, 136.6, 134.9, 130.9, 129.8, 129.3, 128.8, 128.4, 127.5, 126.6, 122.5, 119.3, 80.0, 71.5, 71.4, 71.1, 71.0, 51.7, 40.2, 38.5, 36.7, 24.2, 24.1, 24.1, 24.0, 23.9, 23.8, 23.7, 22.5. ^{31}P NMR (81 MHz, $CDCl_3$) δ 21.3.

DNA extractions, PCR and cloning strategies

Genomic DNA (gDNA) from *Leishmania donovani* was extracted by DNAzol, according to the manufacter's instructions (Invitrogen). The *GDP-MP* gene was amplified from *L. donovani* gDNA by using the following primers: forward 5'-AAC**CATATG**TCTGCATCCGATGGCC-3' (**NdeI**) and reverse 5'-GAT**CTCGAG**CATGATGATCCCAGGC-3' (**XhoI**). *LmGDP-MP* gene was synthesized by GenScript®. The forward primer 5'-AAA**CATATG**TCTGCATCCGATGGCCAGGG-3' (**NdeI**) and reverse primer 5'-CCC**TATAGA**TTACATGATGATCCCAGCC-3' (**XbaI**) were used for *Lm*GDP-MP gene amplification. Each forward and reverse primer include NdeI and XhoI restriction sites (in bold), respectively, except for the reverse primer of *Lm*GDP-MP which presents a XbaI site. The encoding sequence of β2 subunit of *h*GDP-MP was amplified from a cDNA library of Hela S3

cells using AffinityScript QPCR cDNA synthesis kit according to the manufacturer's intructions (Agilent). The primers used for *GDP-MPβ* gene amplification were: forward 5'-AAC**CATATG**AAGGCACTGATCTTAGTGGG-3' (**NdeI**) and reverse 5'-GAT**CTCGAG**CATGAT GATACGAGGCTCTGGC-3' (**XhoI**).

For *Ld*GDP-MP, PCR reaction contained DNA (1 ng), $MgCl_2$ (1 mM), dNTPs (0.4 mM), each primers (2 pmol/μL), and Hot Diamond Taq (1 U; Eurogentec). The PCR was performed with (1) one step of denaturation (95°C for 3 min), (2) 30 cycles of denaturation (95°C for 30 s), - annealing (54°C for 30 s), - elongation (72°C for 1 min 30 s), and (3) one step of extension (15 min at 72°C). Same PCR conditions were used for *LmGDP-MP* and *h*GDP-MP genes, except for the annealing step which was at 55°C. Each amplified fragment was purified by Qiaquick gel extraction kit (Qiagen) and digested with NdeI and XhoI for *Ld*GDP-MP and *h*GDP-MP, or NdeI and XbaI for *Lm*GDP-MP. The digested *LdGDP-MP* and *h*GDP-MP fragments were ligated in the NdeI and XhoI sites of the pET-21b(+) expression vector (Novagen), which encodes a His6 tag fused at the C-terminal end of the protein of interest. *LmGDP-MP* fragment was ligated in the NdeI and XbaI sites of the pnEAvHM expression vector[5,6], which encodes His6 and Maltose Binding Protein (MBP) tags coupled via a TEV (Tobacco etch Virus) cleavage site with the N-terminal part of the protein of interest. Each product of ligation was transformed into DH5α competent cells (Invitrogen) and plasmids of positive clones were then extracted by QIAprep Spin Miniprep kit (Qiagen). The sequence of the each insert was verified on both strands (Eurofins MWG Operon) prior to transformation of *E. coli* BL21(DE3) for expression of recombinant proteins. The plasmid containing the coding sequence of *h*GDP-MP was co-transformed in BL21(DE3) with pG-KJE3 plasmid which contains 5 chaperone genes[7].

Production of recombinant GDP-MPs

A 1/100 dilution of an overnight culture of *E. coli* BL21(DE3) at 37°C was carried out and then cultivated at 37°C until the OD_{600nm} reached 0.6. Protein expression was induced by adding Isopropyl b-D-1-ThioGalactopyranoside (0.5 mM; ITPG) to the medium and bacteria were further cultivated at 30°C for 4 h for *Ld*GDP-MP, or at 15°C overnight for *Lm*GDP-MP and *h*GDP-MP. Since the production of this latter was unsuccessful by using pET21b(+) alone, a co-expression system with chaperone proteins was used to improve the production of *h*GDP-MP[7]. Chaperone protein expression was induced by adding arabinose (0.2%) 30 min prior to IPTG. After protein expression, cells were harvested by centrifugation at 3,300 g for 30 minutes at 8°C and the pellet was resuspended in 40 mL of lysis buffer containing Tris-HCl (50 mM) pH 7.5, NaCl (200 mM), PhenylMethylSulfonyl Fluoride (100 μM; PMSF), Triton X-100 (0.1 %; v/v) and lysozyme (400 μg/mL). The protein extract was then sonicated on ice 4 times for 45 seconds with amplitude 40 and duty cycle 90 (Branson Sonifier 250), and further centrifuged at 20,000 g for 30 min at 8°C. The supernatant was used for further purification.

Purification of recombinant GDP-MPs

All His6 tagged *Ld*GDP-MP, *Lm*GDP-MP and *h*GDP-MP were firstly purified by affinity chromatography by loading the supernatant of protein extracts on columns containing Ni-nitrilotriacetic acid (Ni-NTA) agarose with immobilized nickel ion (Ni-NTA agarose, Qiagen). The columns were then washed with Tris-HCl (50 mM) pH 7.5, NaCl (200 mM; buffer A) containing imidazole (20 mM), and proteins were eluted with buffer A containing from 100 mM to 400 mM imidazole. Each eluted fraction was analyzed by SDS-PAGE followed by Coomassie blue staining and the fractions containing the proteins of interest were concentrated by an Amicon Ultra 5,000 MWCO membrane tube (Millipore) for further purification steps.

*Ld*GDP-MP was then loaded on a size exclusion chromatography Hiload™16/60 Superdex™ 200 column (GE Healthcare), pre-equilibrated with Tris-HCl (50 mM) pH 7.5, NaCl (1 M), DTT (1 mM), glycerol (10 %). This column was connected to a NGC Chromatography System (Bio-rad). Following SDS-PAGE analysis and Coomassie blue staining, the fractions containing the purified *Ld*GDP-MP were then pooled and concentrated at 1.8 mg/ml by an Amicon Ultra 5,000 MWCO membrane tube for further enzymatic assays.

The concentrated fraction of *Lm*GDP-MP coupled with both MBP and His6 tags, was diluted in Tris pH (50 mM) 7.5 to obtain a final concentration of NaCl (50 mM) and injected on a Mono Q anion exchange column (HiTrap Q FF, GE Healthcare) against the TEV cleavage buffer containing Tris-HCl

(50 mM) pH 8, 1 mM DTT, EDTA (0.5 mM), glycerol (5 %). This column was connected to a AKTA purifier (GE Healthcare). The proteins were eluted with a linear gradient of NaCl ranging from 50 mM to 1 M and analyzed by SDS-PAGE followed by Coomassie blue staining. The fractions containing the fusion protein were then pooled and cleaved with TEV (0.28 mg/mL) at room temperature for 3 h, and the product of cleavage was injected in a Hiload™10/300 Superdex™ 200 column (GE Healthcare) pre-equilibrated with Tris-HCl (50 mM) pH 7.5, NaCl (150 mM), DTT (1 mM) in order to remove the TEV and His6-MBP tags. The eluted fractions were further analyzed in SDS-PAGE followed by Coomassie blue staining and the ones corresponding to the peak of interest were pooled, diluted to obtain a final concentration of NaCl (50 mM) and injected on a MonoQ anion exchange column as described above to remove protein contaminants. Following SDS-PAGE analysis and Coomassie blue staining, the fractions containing the purified *Lm*GDP-MP were then pooled and concentrated at 1.7 mg/ml by an Amicon Ultra 5,000 MWCO membrane tube for further enzymatic assays.

Similarly to *Lm*GDP-MP, the concentrated fraction of *h*GDP-MP was diluted in Tris (50 mM) pH 7.5 buffer to obtain a final concentration of NaCl (50 mM) and loaded on a MonoQ anion exchange column against Tris (50 mM) pH 7.5, DTT (1 mM) to remove the chaperone proteins. The proteins were eluted as described above and analyzed by SDS-PAGE followed by Coomassie blue staining. The fractions containing the protein of interest were collected, concentrated by an Amicon Ultra 5,000 MWCO membrane tube (Millipore) and injected on Hiload™ 16/60 Superdex™ 200 column, pre-equilibrated with Tris-HCl (50 mM) pH 7.5, NaCl (200 mM), EDTA (0.5 mM), DTT (1 mM). Following SDS-PAGE analysis and Coomassie blue staining, the fractions containing the purified *h*GDP-MP were then pooled and concentrated at 9.8 mg/ml by an Amicon Ultra 5,000 MWCO membrane tube for further enzymatic assays.

Cell Cultures

Promastigotes of *Leishmania donovani donovani* (MHOM/ET/67/HU3/LV9) and *Leishmania mexicana mexicana* (MNYC/BZ/62/M379) were cultured in the dark at 26ºC with 5% CO_2 in M199 complete medium containing M199 medium supplemented with adenosine (100 µM), hemin (0.5 mg/L), Hepes (40mM) pH 7.4 and heat inactivated foetal bovine serum (10 %; HIFBS). Cultures of axenic amastigotes of *L. donovani* was adapted from[8]. Briefly, axenic amastigotes of *L. donovani* were obtained from late log promastigotes diluted at 1 x 10^6/mL in M199 complete medium acidified at pH 5.5 and cultured at 37°C with 5% CO_2. Axenic amastigotes of *L. mexicana* were obtained from late log promastigotes diluted at 1 x 10^6/mL in Schneider's insect medium supplemented with HIFBS (20%), hemin (18.75 µg/mL), pH 5.5 and cultured at 32°C with 5% CO_2[9].

The RAW 264.7 macrophages (from ATCC) were cultured at 37°C with 5% CO_2 in DMEM complete medium containing Dulbecco's Modified Eagle's Medium (DMEM, Invitrogen) supplemented with penicillin-streptomycin (100 U/mL; Invitrogen), and heat-inactivated fetal bovine serum (10 %; HIFBS).

Bone marrow derived macrophages (BMDM) were obtained from female Balb/c mice (Janvier), from 8- to 12-week of age, as previously described[10]. BMDM were further cultured in DMEM complete medium at 37°C with 7% CO_2.

All *E. coli* cultures were carried out in Luria-Bertani (LB) medium (Thermofisher) supplemented with ampicillin (100 µg/mL) when transformed with pET21b(+) or pNEAvHM expression vectors, and with chloramphenicol (50 µg/mL) when pGKJE3 plasmid is contained in bacterial cells.

Molecular docking

The protein structures of *Ld*GDP-MP and *h*GDP-MP were generated by homology modeling, and relaxed using all-atom molecular dynamics (MD) simulations in explicit solvent, as previously described[2]. Then, the most populated conformation of each GDP-MP was identified by MD trajectory clustering analyses, and was used for the present docking study. Three ligands were docked on the GDP-MP models: compounds **99** and **100** and GDP-mannose. Their initial three-dimensional structures were generated using the program MarvinSketch 6.2.1 from ChemAxon (http://www.chemaxon.com). All docking calculations were performed using the AutoDock Vina program[11], with a search box of volume 30 x 25 x 25 $Å^3$ and centered around the GDP-Mannose position in the *L. donovani* GDP-MP model. The docking of each ligand on each protein were

performed 10 times and generated, in all, 100 binding modes which were further analyzed. To identify the most probable complex conformations, we visually inspected the 100 lowest energy poses of each compound in the GDP-MP catalytic site and, based on bioisostere considerations, we selected those which have their quinoline group located in the same place as the guanine group of the GDP-Mannose. From the counting of these most plausible poses, the docked inhibitor potency, relatively to the natural substrate GDP-Mannose, can be qualitatively estimated.

References

1. Pomel, S., Rodrigo, J., Hendra, F., Cavé, C., and Loiseau, P.M. *In silico* analysis of a therapeutic target in *Leishmania infantum*: the guanosine-diphospho-D-mannose pyrophosphorylase. *Parasite* **19,** 63-70 (2012).
2. Daligaux, P. *et al.* Comparative study of structural models of *Leishmania donovani* and human GDP-mannose pyrophosphorylases. *Eur. J. Med. Chem.* **107,** 109-118 (2016a).
3. Daligaux, P., Pomel, S., Leblanc, K., Loiseau, P.M., & Cavé, C. Simple and efficient synthesis of 5'-aryl-5'-deoxyguanosine analogs by azide-alkyne click reaction and their antileishmanial activities. *Mol. Divers.* **20,** 507-519 (2016b).
4. Lackovic, K. *et al.* Inhibitors of *Leishmania* GDP-mannose pyrophosphorylase identified by high-throughput screening of small-molecule chemical library. *Antimicrob. Agents Chemother.* **54,** 1712-1719 (2010).
5. Diebold, M.L., Fribourg, S., Koch, M., Metzger, T., & Romier, C. Deciphering correct strategies for multiprotein complex assembly by co-expression: Application to complexes as large as the histone octamer. *J. Struct. Biol.* **175,** 178-188 (2011).
6. Haffke, M. *et al.* Characterization and production of protein complexes by co-expression in *Escherichia coli*. *Methods Mol. Biol.* **1261,** 63-89 (2015).
7. Nishihara, K., Kanemori, M., Kitagawa, M., Yanagi, H., & Yura, T. Chaperone coexpression plasmids: differential and synergistic oles of DnaK-DnaJ-GrpE and GroEL-GroES in assisting folding of an allergen of Japanese cedar pollen, Cryj2, in *Escherichia coli*. *Appl. Environ. Microbiol.* **64,** 1694-1699 (1998).
8. Saar, Y. *et al.* Characterization of developmentally regulated activities in amastigotes of *Leishmania donovani*. *Mol. Biochem. Parasitol.* **95,** 9-20 (1998).
9. Bates, P.A., Robertson, C.D., Tetley, L., & Coombs, G.H. Axenic cultivation and characterization of *Leishmania mexicana* amastigote-like forms. *Parasitology* **105,** 193-202 (1992).
10. Aulner, N., *et al.* High content analysis of primary macrophages hosting proliferating *Leishmania* amastigotes: application to anti-leishmanial drug discovery. *PLoS Neglect. Trop. Dis.* **7,** e2154 (2013).
11. Trott, O., & Olson, A.J. AutoDock Vina: improving the speed and accuracy of docking with a new scoring function, efficient optimization, and multithreading. *J. Comput. Chem.* **31,** 455-461 (2010).
